# Raman signatures of *Cnm*-positive *Streptococcus mutans*: II, screening the virulence of clinical isolates

**DOI:** 10.3389/fmicb.2026.1784126

**Published:** 2026-04-22

**Authors:** Giuseppe Pezzotti, Tetsuya Adachi, Kazunori Kitagawa, Saki Ikegami, Hayata Imamura, Toshiro Yamamoto, Kazu Okuma, Yoshiyuki Matsuo, Wenliang Zhu, Yoshiki Yasukochi, Koichiro Higasa, Saki Nishihama, Katsuhiro Takeda, Hideki Shiba, Miki Kawada-Matsuo, Hitoshi Komatsuzawa

**Affiliations:** 1 Biomedical Engineering Center, Kansai Medical University, Osaka, Japan; 2 International Center for Biomedical Industrial Promotion, Kansai Medical University, Osaka, Japan; 3 Department of Immunology, Graduate School of Medical Science, Kyoto Prefectural University of Medicine, Kyoto, Japan; 4 Department of Orthopedic Surgery, Tokyo Medical University, Tokyo, Japan; 5 Department of Molecular Science and Nanosystems, Ca’ Foscari University of Venice, Venice, Italy; 6 Biomarker Disease Laboratory, IRCCS San Camillo Hospital, Venice, Italy; 7 Department of Orthopaedic Surgery, Mie University, Graduate School of Medicine, Mie, Japan; 8 Department of Dental Medicine, Graduate School of Medical Science, Kyoto Prefectural University of Medicine, Kyoto, Japan; 9 Department of Microbiology, Kansai Medical University, School of Medicine, Osaka, Japan; 10 Ceramic Physics Laboratory, Kyoto Institute of Technology, Kyoto, Japan; 11 Department of Genome Analysis, Institute of Biomedical Science, Kansai Medical University, Osaka, Japan; 12 Central Research Center, Institute of Biomedical Science, Kansai Medical University, Osaka, Japan; 13 Department of Biological Endodontics, Graduate School of Biomedical & Health Sciences, Hiroshima University, Hiroshima, Japan; 14 Department of Bacteriology, Graduate School of Biomedical & Health Sciences, Hiroshima University, Hiroshima, Japan

**Keywords:** clinical isolates, Cnm protein, Raman algorithms, *Streptococcus mutans*, virulence

## Abstract

This study dealt with developing a Raman spectroscopic method for estimating the degree of virulence of *Streptococcus mutans* bacteria isolated from clinical swab samples. Raman experiments aimed at establishing suitable spectroscopic parameters to quantify bacterial virulence and were conducted on a limited series of six clinical isolates three of which were genomically classified as Cnm-positive and three as Cnm-negative. Samples were characterized after biofilm purification and compared with cultures of the same bacteria in physiological state of equilibrium, namely, after long-term stabilization *in vitro*. Statistically significant series of ten Raman spectra were collected at different locations on each clinical sample, and their averages interpreted as multiomic snapshots of bacterial structure. Building upon the spectroscopic analyses described in the companion paper Part I, Raman characterizations of clinical isolates revealed a significant degree of variability in the bacterial structure, but also suggested clear classification criteria for clinical samples. These spectroscopic criteria reflected specific biochemical circumstances affecting the structure of bacteria in their pathophysiological state. Raman algorithms based on the fractional balance between proteins and peptidoglycans, and the degree of protein structural disorder vs. presence of oxysulfur compounds enabled insightful classifications of bacterial virulence, which matched genomic analyses. These structural characteristics, which allowed distinguishing between Cnm-positive and Cnm-negative bacteria, could provide fast and unbiased diagnostic criteria for risk assessments of endocarditis and hemorrhagic strokes as induced by Cnm-positive bacteria. In summary, the present study proposes a new spectroscopic approach to oral flora-related diagnostics and confirms the potential utility of Raman spectroscopy in chairside analyses of clinical isolates.

## 1 Introduction

Cnm-positive is a specific class of *Streptococcus mutans* strains, a common bacterium primarily known for its role in tooth decay (Loesche, 1986; Nguyen et al., 2022). The *Cnm* gene encodes collagen-binding adhesins, which allow the bacterium to promptly bind to collagen-containing tissues in the human body, including heart, blood vessels, and bones (Aviles-Reyes et al., 2014). Similarly, but more aggressively with respect to Cnm-negative *Streptococcus mutans* strains, Cnm-positive bacteria induce tooth decay upon setting off a severely acidic environment, which quite effectively erodes the enamel structure. However, besides enabling more aggressive forms of tooth decay, its ability to strongly adhere to soft tissues represents by far the most severe concern for human health, beyond dental issues; possibly related pathologies including endocarditis (Mylonakis and Calderwood, 2001; Nagata et al., 2006; Nakano et al., 2008), atherosclerosis (Nakano et al., 2006; Kesavalu et al., 2012), hemorrhagic strokes (Nakano et al., 2011; Mansour et al., 2017), osteomyelitis (Ullman et al., 1988; Murillo et al., 2014), and septic arthritis (Parra-Grande et al., 2019).

Dental procedures (e.g., tooth extractions or gum disease treatments), or even daily activities like brushing or flossing, could facilitate the introduction of *Streptococcus mutans* bacteria into the bloodstream. Once in the bloodstream, the bacteria might travel to various parts of the body, including heart and brain. In such circumstances, Cnm-positive strains are more likely to cause bacteremia, because they can more effectively adhere to soft tissues. Individuals with pre-existing heart conditions (e.g., damaged heart valves, prosthetic valves, or congenital heart defects) are particularly susceptible to infective endocarditis, because damaged heart tissue exposes collagen, thus providing ideal binding/accumulation sites for Cnm-positive strains. High blood pressure is another factor favoring bacterial adhesion, because the enhanced pressure widens dehiscent junctions between endothelial cells and exposes the tunica intima, namely, the innermost layer of the veins, to which bacteria adhere most easily. Fragments of bacteria and biofilm fibrin can then break off and travel to other parts of the body, causing emboli in major organs, such as brain, kidneys, or lungs, and thus resulting in stroke and other organs’ failure (Stinson et al., 1984; Tonomura et al., 2016; Inchaustegui et al., 2018; Inenaga et al., 2018; Ito et al., 2019; Hosoki et al., 2022; Tonelli et al., 2023). In view of the above, understanding the chemical details of *Streptococcus mutans* pathophysiology clearly extends far beyond oral health.

In the companion Part I paper (Pezzotti et al., 2026a), we compared statistically significant average Raman spectra collected on long-term *in vitro*-cultured *Streptococcus mutans* strains expressing the *cnm* gene in comparison to the same bacteria lacking that gene. A number of striking structural differences were found at the molecular scale, which provided a conceptual path to explain the molecular origin of cerebral microbleeds, namely, the copious presence of residual oxysulfur molecules in the Cnm-positive strain, in fractions that conspicuously lacked in the Cnm-negative one. Vibrational fingerprints pointed to indoxyl and other oxysulfur molecules, thus suggesting that the Cnm-positive strains exploit sulfur chemistry in anchoring to soft tissues. This structural peculiarity paired with a significant increase in the amount of highly disordered protein structures (i.e., wrapping the bacterial cells) and a clear simplification of the peptidoglycan membrane structure. These bold molecular characteristics link microbial structure to human diseases and suggest a new Raman spectroscopic path towards quantitative oral flora diagnostics for personalized therapeutic interventions.

In particular, the presence of oxysulfur molecules on the bacterial surface can potentially increase bacteria’s adhesiveness to soft tissues. Oxysulfur groups, such as sulfonic (–SO_3_H) or sulfinic (–SO_2_H) acids, are polar and can form hydrogen bonds or strong electrostatic interactions with biological tissues, which are often composed of glycoproteins and other molecules that have hydroxyl (–OH) and amine (–NH_2_) functional groups. These interactions can greatly enhance the ability of bacteria to adhere to soft tissues, contributing to their colonization and potential pathogenicity. In addition, oxysulfur-containing proteins could alter the surface charge and hydrophilicity of bacterial cell walls, further influencing their interactions with host tissues. A number of multifactorial and synergic adhesion mechanisms other than oxysulfur chemistry have so far been reported for different bacteria (Kreve and Dos Reis, 2021). They typically involve surface proteins (adhesins) and/or polysaccharides, so that the full extent of their contribution generally depends on the specific bacterial species and environment (Takahashi et al., 2013; Berne et al., 2018; Straub et al., 2019; Wang et al., 2019; Kreve and Dos Reis, 2021). While it is generally recognized that bacteria utilize a wide range of chemical and mechanical mechanisms to sense their environment and to survive hostile conditions, works citing exploitation of oxysulfur chemistry in enhancing adhesion are seldom found in the microbiology literature of *Streptococcus mutans*. In a remarkably innovative set of mechano-microbiological experiments by atomic force microscopy, Wang C. et al. (2020) were able to measure the adhesion forces of *Streptococcus mutans* strains to various surfaces. Those researchers precisely located the spatiotemporal conditions for improved adhesiveness, while also linking adhesion forces to gene expression in the presence of other streptococcal species acting as initial colonizers. Building upon that understanding, we newly suggest here that Cnm-positive strains are self-sufficient in their adhesive functionality. Since necessitating strong adhesion during fast traveling inside the human cardiovascular system, they altered their structure to exploit an oxysulfur “glue” as a powerful chemical weapon.

In this paper, we build upon the finding of oxysulfur chemistry-related enhancement in adhesiveness for Cnm-positive strains previously described in the companion Part I paper, and look for quantitative parameters to be extracted from the Raman spectrum, which could assess the degree of adhesiveness (and thus the intrinsic virulence) in clinical isolates. For doing so, we examined a series of six clinical isolates, half of which were preliminary classified by means of genomic analyses as Cnm-positive [*Cnm*^(+)^*Sm*, henceforth] and half as Cnm-negative [*Cnm*^(−)^*Sm*]. Establishing spectroscopic parameters to screen in a quantitative way the virulence of *Streptococcus mutans* clinical isolates is seen here as a propaedeutic step to a large-scale set of double-blind genomic/spectroscopic analyses performed on a larger number of clinical samples. These additional analyses are presented in the companion Part III paper (Pezzotti et al., 2026b) and enable to finally substantiate the statistical relevance of the novel Raman methodology.

## 2 Experimental procedures

### 2.1 Isolation of *Streptococcus mutans*

*Streptococcus mutans* (*S. mutans*) was isolated from the oral cavity of the outpatients who visited the Department of Neurology of Hiroshima University Hospital. Sterile cotton swabs (Kawamoto Mekkin Swab #104, Hydraflox Swab 25-3706-H) were used to collect samples from the oral mucosa (i.e., buccal mucosa, tongue, palate, and oral vestibule). Cotton swab was streaked on Mitis-Salivarius agar medium (Beckton Dickinson Microbiology Systems) containing bacitracin (final 32 μg/mL) (MSB) and incubated for 2 days at 37 °C with 5% CO_2_. The colonies grown on MSB agar were picked and re-streaked on trypticase soy broth (TSB) (Becton, Dickinson and Company). After incubation for 1 day at 37 °C with 5% CO_2_, a small portion of bacterial cells was suspended in 100 μL of lysis buffer (0.01% Triton X-100 in Tris-EDTA buffer; pH 8.0). After heating at 95 °C for 10 min, bacterial suspension was centrifuged at 10,000 × g for 5 min. After centrifugation, the supernatant was directly used for DNA template for polymerase chain reaction (PCR), resulting in successful amplification. Using *S. mutans* specific primers (R: GCCATACACCACTCATGAATTGA, F: GTCTACAGCTCAGAGATGCTATTCT), PCR was first performed to verify that the strains actually consisted of *S. mutans*. Then, the isolated *S. mutans* strains were checked with respect to their *cnm* gene by means of PCR using the specific primers for *cnm* (R: GTGGATCCAGCAGTAACATTTCATCG5, F: TTAAGCTTAGCTACAGAATGGTTAAATTC).

*Streptococcus mutans* isolation was approved by the ethics committee of the Hiroshima University Hospital Review Board (approval number E2021-2581). All methods were performed in accordance with the approved guidelines and regulations.

### 2.2 Whole genome sequence analysis

*Streptococcus mutans* chromosomal DNA was extracted according to a method described elsewhere (Shibata et al., 2003). Briefly, 2 mL of *S. mutans* culture grown in TSB for 12 h were first collected. Then, bacterial cells were suspended in 0.5 mL CS buffer (100 mM Tris–HCl [pH 7.5], 150 mM NaCl, 10 mM EDTA) containing 10 μL of mutanolysin (Sigma-Aldrich, St. Louis, MO, USA) (5 mg/mL) and 5 μL of RNase (Nippon Gene, Tokyo, Japan) (10 mg/mL). After incubation at 37 °C for 10 h, proteinase K (Nacalai Tesque, Kyoto, Japan) (10 mg/mL) and SDS (final 1%) were added, followed by incubation at 55 °C for 12 h. Following a treatment with phenol and, successively, by phenol-chloroform, DNA was precipitated by ethanol. Precipitates were dissolved in sterilized deionized water. Whole genome sequences (WGS) were performed by Illumina HiSeq X Ten platform. The Illumina read data for each isolate were used for *de novo* assembly using Shovill v1.1.0 Seemann T. Shovill: faster SPAdes assembly of Illumina 2018.^1^ The genome data of the isolates in this study have been deposited in the DDBJ BioProject database as accession no. PRJDB19019.

The serotypes of *S. mutans* was determined in previously published literature (Shibata et al., 2003; Nakano et al., 2004): rgpH (Accession No. AB108684) for serotype c, ORF3e (Accession No. AB108685) for serotype e, ORF2f (Accession No. AB108686) for serotype f, and the 5′ region of the rgpF gene (350 bp from the initial sequence; modified from Accession No. AB010970) for serotype k as reference gene. To verify the virulence-related genes, genes coding Protein antigen from serotype c (PAc), Cnm protein and 3 glucosyltransferases (*gtfB*, *gtfC*, *gtfD*) were searched in the National Centre for Biotechnology Information (NCBI) database. The obtained *PAc* (Accession no. AB548069.1), *Cnm* coding gene (Accession no. AB102689.1), *gtfB*, *gtfC* and *gtfD* (Accession no. NC_004350.2) were used as reference genes.

### 2.3 Immunostaining and fluorescence imaging

24 h-cultured *S. mutans* bacteria were fixed in 4% paraformaldehyde (PFA) for 10 min at room temperature (RT) and permeabilized with permeabilization buffer (1X PBS/3% FBS/0.05% Triton X-100) for 30 min at RT. The cells were then washed three times at RT and stained overnight with anti-*S. mutans* (mouse) antibody (final concentration: 10 μg/mL) at 4 °C. Cells were finally washed with a permeabilization buffer and incubated with POLARIC-500BCS (Goryo Chemical, Inc., Chuo-city, Sapporo, Japan), goat anti-mouse IgG PE (1: 200) (Tokyo Chemical Industry Co. Ltd., Chuo-city, Tokyo, Japan), and DAPI stain (1: 500) (DOJINDO LABORATORIES, Kamimashiki-gun, Kumamoto, Japan) for 60 min under mild agitation at RT in the dark. The POLARIC (green) stained the membrane, and the DAPI (blue) stained the nucleus, while the anti-*S. mutans* (red) antibodies recognized proteins on the bacterial surface. The concurrent appearance of red and green fluorescence eventually led to a violet mark on the cell surface. The iCBiofilm-H2 (Tokyo Chemical Industry Co. Ltd.) was used to conduct biofilm clearing, according to manufacturer instructions. Staining was observed under a fluorescence microscope BZX810 (Keyence, Osaka, Japan) and a confocal microscope AX-R with NSPARC (Nikon Corporation, Tokyo, Japan).

### 2.4 Raman spectroscopy and machine learning algorithms

Raman spectra were collected *in situ* on living *Streptococcus mutans* bacterial samples collected from swab samples of six different patients. The samples were first cleaned up of their biofilms and genomically analyzed to classify them into two distinct categories: *Cnm*^(+)^*Sm* and *Cnm*^(−)^*Sm*. Bacterial samples were analyzed after 2 days culture both in presence and in absence of sucrose. Raman data were collected by means of a high spectrally resolved spectrometer designed for measurements on biological samples (LabRAM HR800, Horiba/Jobin-Yvon, Kyoto, Japan). The equipment launched an optical circuit (set in confocal mode with a 20x objective lens) employing a holographic notch filter, which concurrently enables high signal efficiency and high spectral resolution. The wavelength of the incoming light was 532 nm, as generated by a solid-state laser source operating at 10 mW. The Raman scattered light was monitored by means of a single monochromator interfaced with an air-cooled charge-coupled device (CCD) detector (Andor DV420-OE322; 1024 × 256 pixels). The acquisition time for a single spectrum was typically 10 s for three successive acquisitions at each location. A spectral resolution of better than 1 cm^−1^ was achieved by concurrently collecting (at each measurement) an internal reference signal from a selected neon lamp to calibrate the spectrometer. Series of 10 spectra were systematically collected at different locations (over a total area of ~2 mm^2^) for each bacterial sample and averaged in order to obtain statistically representative spectra. Raman spectra were compared with reference *Cnm*^(+)^*Sm* and *Cnm*^(−)^*Sm* strains cultured without sucrose and long-term stabilized. These latter strains, described in detail in the companion Part I paper (Pezzotti et al., 2026a), will henceforth be referred to as “equilibrium” strains.

Experimental Raman spectra were subjected to polynomial baseline subtraction and deconvolution into series of Gaussian-Lorentzian sub-band components. The baseline subtraction procedure was performed using options available in commercial software (LabSpec 4.02, Horiba/Jobin-Yvon, Kyoto, Japan) with fixed criteria for all collected spectra. All spectra were analyzed after intensity normalization to the strongest signal in the collected spectral interval. Detailed descriptions of spectral deconvolution criteria have been reported in previously published papers (Pezzotti, 2021; Pezzotti et al., 2021b). An automatic solver exploiting a linear polynomial expression of Gaussian-Lorentzian functions was iteratively run to match average experimental spectra for minimum scatter (better than 95% confidence interval) with the experimental (average) spectrum. It is important to strengthen here that the algorithm employed here for spectral deconvolution is based on a selection of signals belonging to real molecules and not just on a mere mathematical criterion. It relies on conditions imposed on band positions, relative intensity, and bandwidths provided the required mathematical constraints to univocally deconvolute the experimental spectra. In the present context, it should be noted that a spectral deconvolution is intrinsically an ill-posed inverse problem, and purely mathematical fitting procedures do not guarantee a unique solution, as multiple combinations of unconstrained bands may reproduce the experimental spectrum with comparable statistical quality. In contrast, deconvolution constrained by a reference library of Raman spectra from well-characterized elementary molecules introduces physically and chemically meaningful boundary conditions. Upon embedding molecular-scale information into the fitting procedure, this approach reduces solution degeneracy and enables a structurally consistent and chemically interpretable reconstruction of the experimental spectrum.

### 2.5 Statistical analyses

The statistical relevance of the parameters extracted from the Raman experiments was analyzed by computing mean values and standard deviations. Statistical validity was evaluated by applying the unpaired Student’s *t*-test. Values *p* < 10^−2^ and *p* < 10^−3^ were considered as statistically significant and labeled with two and three asterisks, respectively.

## 3 Experimental results

### 3.1 Identification of virulence genes

The whole genome sequence of the six studied *S. mutans* isolates were analyzed to identify their serotypes, etiology, and virulence factors. We focus here on the structural genes responsible for protein antigen from serotype c (*PAc*), glucosyltransferases (*gtf*s), and *cnm*. *PAc* was reported as a major adhesin to pellicles (derived salivary components) on the tooth surface (Okahashi et al., 1989; van Houte, 1994; Watanabe et al., 2021); *gtf*s are associated with the synthesis water-insoluble sticky glucan responsible for dental plaque formation (Bowen and Koo, 2011); and, *Cnm* is a collagen-binding protein, which has been reported to be associated with cerebral microbleeds (Avilés-Reyes et al., 2016; Hosoki et al., 2020). Regarding serotypes, 5 strains (i.e., HST12, 17, 28, 23 and 40) only contained genes for serotype c, while HST62 showed serotype c and k double positive. All strains possessed the *PAc* gene and three *gtf*s, namely, *gtfB*, *gtfC*, and *gtfD*, while the *cnm* gene was only found in 3 strains (HST23, HST40 and HST62). The results of whole genome sequence analysis are summarized in Supplementary Table 1.

### 3.2 Confocal fluorescence imaging

**Figure 1:**
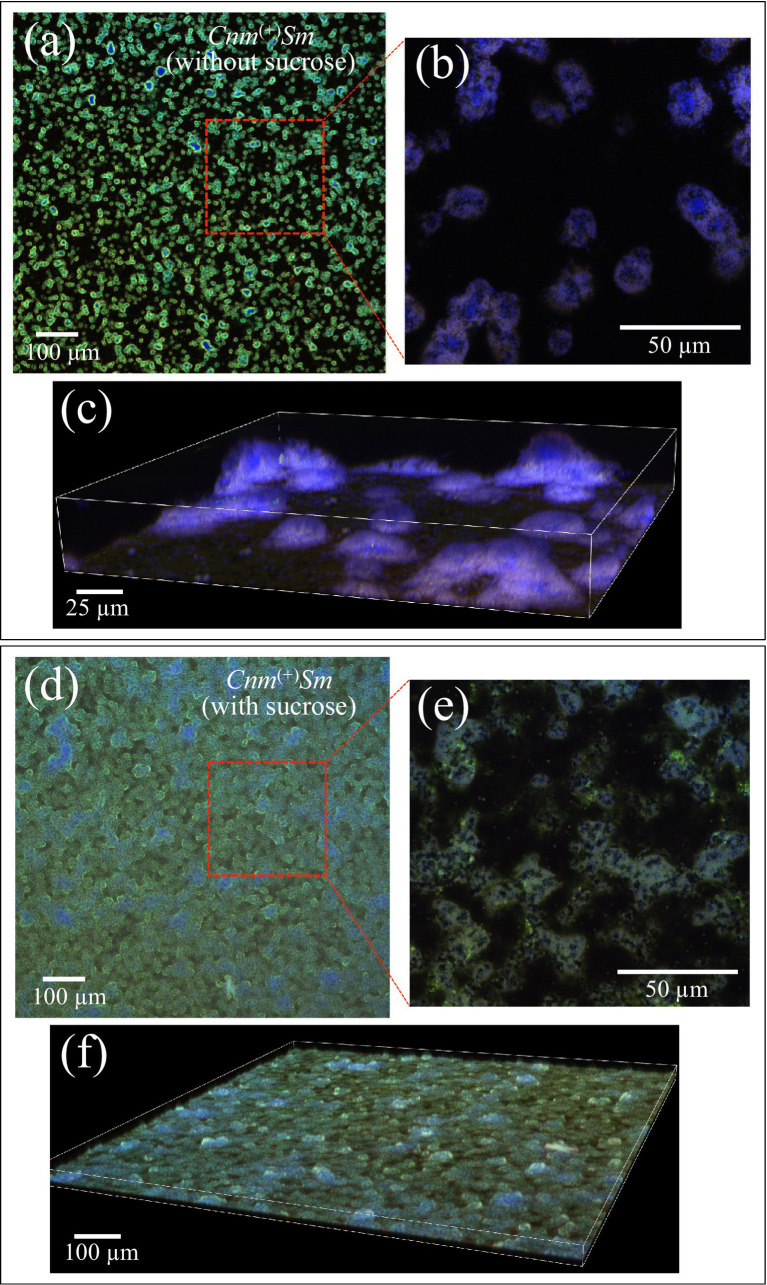
In **(a–c)**, fluorescence and confocal microscopy images of the *Cnm*^(+)^*Sm* HST-23 isolate cultured for 1 day without supplying sucrose; a similar characterization if the same isolate cultured under the same conditions, but with supplying sucrose, is shown in **(d–f)**. Green and blue pixels represent cells’ membrane and nuclei moieties, respectively (cf. Section 2.3). The red stain, associated with proteins on the bacterial surface, overlaps with the green pixels from the bacterial membrane and displays violet.

Figures 1a–c show fluorescence and confocal fluorescence microscopy images of *Cnm*^(+)^*Sm* isolate (specifically the sample HST-23) cultured for 1 day without supplying sucrose. Green and blue pixels give cells’ membrane and nuclei moieties, respectively (cf. Section 2.3). Note that the red stain, associated with proteins on the bacterial surface, in overlap with the green pixels from the bacterial membrane, displays as violet in Figures 1b,c. As seen, the bacterial cells appeared almost completely separated from each other (cf. black areas of empty volumes). On the other hand, culturing the same bacteria with supplying sucrose led to a copious formation of biofilm, so that the bacterial cells conspicuously stacked to each other’s, as shown in the microscopy images in Figures 1d–f. The same imaging procedures shown above were performed on all investigated isolates and gave, as far as the mere sample morphology was concerned, very similar results, independent of the isolate belonging of *Cnm*^(+)^*Sm* or *Cnm*^(−)^*Sm* families. In the remainder of this paper, cultures both without and with sucrose addition will be analyzed for all bacterial isolates in order to assess the molecular compositions of bacterial cells and of their biofilms, respectively.

### 3.3 Raman spectra of clinical isolates

**Figure 2:**
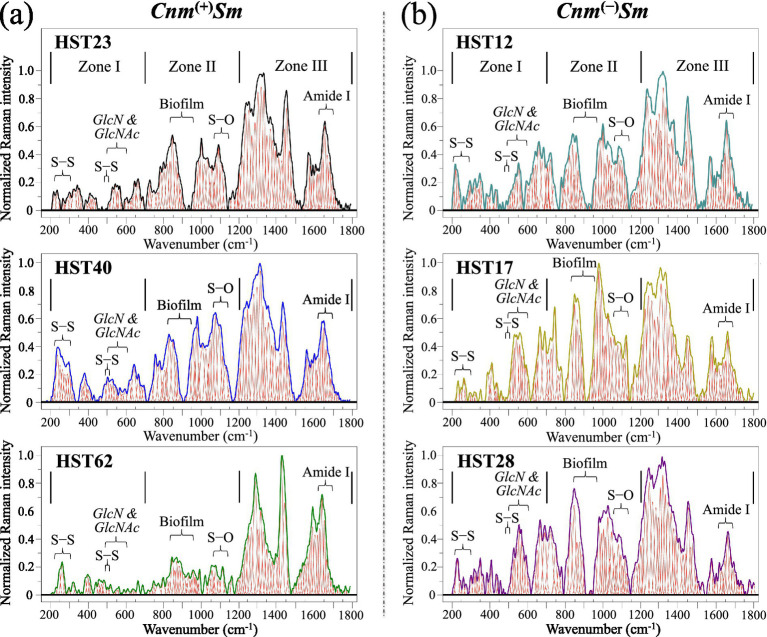
Raman spectra in the wavenumber interval 200–1800 cm^−1^ on **(a)** *Cnm*^(+)^*Sm* and **(b)** *Cnm*^(−)^*Sm* clinical isolates (cf. sample names in inset) after biofilm cleaning procedure and successive culture for 24 h in absence of sucrose. Each spectrum is an average of ten spectra collected at different locations (over a total area of ~2 mm^2^). Spectra are divided into three characteristic zones at low, intermediate, and high wavenumbers. Labels in inset locate chemical bonds and other molecular features discussed in detail in the remainder of the paper (*GlcN* and *GlcNAc* are abbreviations for glucosamine and *N*-acetylglucosamine, respectively).

Figure 2 shows average Raman spectra collected in the wavenumber interval 200–1800 cm^−1^ on (a) *Cnm*^(+)^*Sm* and (b) *Cnm*^(−)^*Sm* clinical isolates (cf. sample names in inset) after undergoing biofilm cleaning procedure and successive culture for 24 h in absence of sucrose. Each spectrum is the average of 10 spectra collected at different locations (over a total area of ~2 mm^2^). Since cultured in absence of sucrose, all samples lacked biofilm formed during culture, however they yet contained some biofilm residues stemming from the original clinical samples. All spectra were deconvoluted into series of Gaussian-Lorentzian sub-bands and divided into three main wavenumber sub-zones, henceforth referred to as Zones I, II, and III in the intervals 200–700, 700–1200, and 1200–1800 cm^−1^, respectively. As seen from Figure 2, the morphology of all spectra presented strong morphological similarities, but clearly different patterns also appeared in comparing spectra from *Cnm*^(+)^*Sm* and *Cnm*^(−)^*Sm* clinical isolates. Such spectral variations pointed to bold structural differences in molecular structure, which will be discussed in detail in the next section. In inset to each spectrum, specific wavenumber intervals are emphasized with reference to signals related to the key molecules that will be attentioned in the present analysis (cf. labels in inset to Figure 2). In particular, Zone I is characteristic for S–S stretching and other S-bond-related bands from a variety of sulfur compounds (225–370 cm^−1^ and 440–450 cm^−1^) (Sato et al., 1985); Zone I also encompasses a series of skeletal-mode signals from glucosamine (*GlcN*) and *N*-acetylglucosamine (*GlcNAc*) molecules (between 480 and 620 cm^−1^) (She et al., 1974). These signals arise from peptidoglycan structures in the external membrane of gram-positive bacteria (Kim et al., 2015). Zone II contained stretching signals from S–O bonds in oxysulfur molecules, which are comprises in the wavenumber interval 1050–1125 cm^−1^ (Sato et al., 1985). In addition, Raman bands of glucan structures from biofilm residues belonging to the original clinical samples could also be spotted in all isolates (890–950 cm^−1^) (Wiercigroch et al., 2017). Finally, in Zone III, the most important feature was represented by the Amide I mode, which characterizes the secondary structure of proteins (cf. wavenumber interval 1630–1700 cm^−1^) (Rygula et al., 2013).

The above key-features contain important hints about the molecular structure of different clinical isolates, which should in turn reflect their different pathological impact on human health. A detailed analysis of spectral differences among *Cnm*^(+)^*Sm* and *Cnm*^(−)^*Sm* clinical isolates is offered in the next section. Such analysis will clarify fundamental metabolomic differences between the two genomically different isolate categories.

### 3.4 Raman spectra in specific wavenumber intervals

In this section, averaged and deconvoluted experimental spectra from different clinical isolates are examined in detail by analyzing high spectrally resolved wavenumber regions in the above-mentioned Zones I–III. Looking for specific features to distinguish between *Cnm*^(+)^*Sm* and *Cnm*^(−)^*Sm* isolates, the characteristic features of these spectral zones are compared with those of long-term cultured “equilibrium” strains of the same bacteria, whose spectra were reported and discussed in detail in the companion Part I paper (Pezzotti et al., 2026a).

The subdivision of the Raman spectrum into three main spectral regions (200–700 cm^−1^, 700–1200 cm^−1^, and 1200–1800 cm^−1^) was performed to enable a chemically meaningful and functionally oriented interpretation of the biochemical differences among different strains, rather than representing an arbitrary segmentation. The 200–700 cm^−1^ region (Zone I) corresponds to the low-wavenumber “fingerprint” domain dominated by skeletal and heavy-atom vibrations, where S–S and C–S stretching modes, as well as oxysulfur-related vibrations can be selectively observed with minimal interference from intense carbohydrate and amide bands. This region is particularly sensitive to disulfide bridges and sulfur-containing membrane components, allowing us to probe structural differences in membrane-associated molecules. Importantly, in this same window glycogen and key *GlcN*/*GlcNAc* skeletal deformations also appear, enabling a direct internal comparison between sulfur chemistry and carbohydrate-associated structural motifs that are central to cell wall and extracellular matrix organization. The 700–1200 cm^−1^ region (Zone II) encompasses the main polysaccharide fingerprint bands, including C–O, C–C, and C–O–C stretching vibrations characteristic of biofilm exopolysaccharides, as well as S–O stretching modes that corroborate the oxysulfur features detected in Zone I. Upon examining this region, one could both quantify biofilm-associated carbohydrate content and validate sulfur oxidation states inferred from low-frequency modes, thus strengthening the chemical assignment through cross-region consistency. Additionally, *GlcNAc* ring vibrations in this interval provide complementary and independent structural information to that discussed in Zone I, improving interpretative robustness. Finally, the 1200–1800 cm^−1^ region (Zone III) contains the Amide I and Amide III bands, which are highly sensitive to protein secondary structure (*α*-helix, *β*-sheet, random coil). Focusing on this window allows one to compare conformational differences in membrane-associated proteins among strains. Because protein secondary structure strongly influences bacterial adhesion, stress response, and biofilm maturation, analyzing this region provides functional context to the membrane and polysaccharide differences identified in Zones I and II. Therefore, the tripartite division reflects three chemically and biologically distinct yet complementary domains: (i) sulfur chemistry and membrane skeletal features, (ii) polysaccharide and sulfur-oxide confirmation within the biofilm matrix, and (iii) protein secondary structure. This structured approach enhances interpretability, reduces spectral overlap bias, and allows mechanistic correlations between membrane composition, extracellular matrix architecture, and protein conformation in different isolates.

#### 3.4.1 Zone I—low wavenumber region (200–700 cm^−1^)

**Figure 3:**
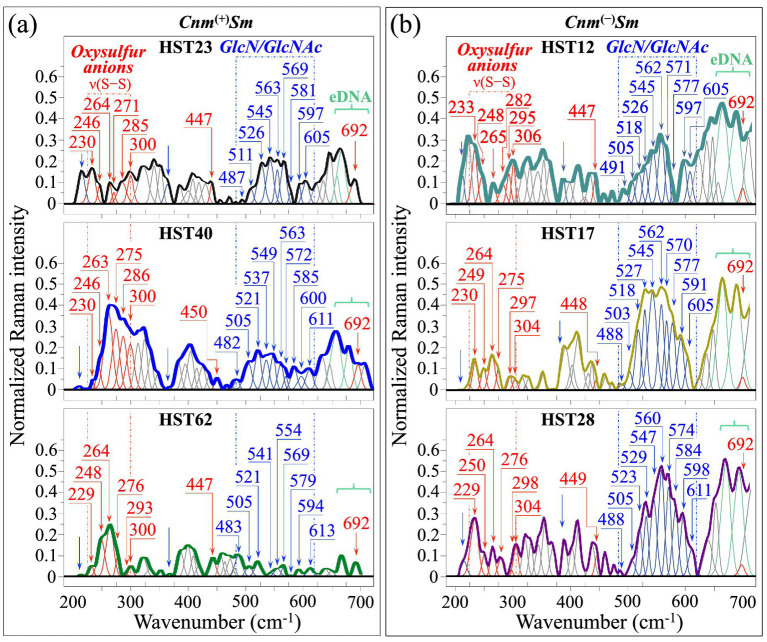
High spectrally resolved (average) Raman spectra collected on **(a)** *Cnm*^(+)^*Sm* and **(b)** *Cnm*^(−)^*Sm* isolates (cf. sample names in inset); bands related to oxysulfur anions, *GlcN*/*GlcNAc*, and extracellular DNA (eDNA) are emphasized in red, blue, and light green, respectively. The meaning of two additional blue arrows is given in text. Wavenumbers in correspondence of each band are given in cm^−1^.

Figures 3a,b show high spectrally resolved (average) Raman spectra collected on *Cnm*^(+)^*Sm* (HST23, HST40, and HST62; cf. labels in inset) and *Cnm*^(−)^*Sm* (HST12, HST17, and HST28; cf. labels in inset) isolates, respectively. The presence of S–S bond stretching and other S-bond-related vibrational modes in this wavenumber region contributes six distinct, but partly overlapping signals, in the interval 229–306 cm^−1^, and one additional signal at 447–450 cm^−1^ (cf. sub-bands in red color and labels in inset to Figure 3). The sulfur-containing molecules responsible for the above-mentioned six signals in Zone I could be identified, as follows (Sato et al., 1985): tetrathionate ions (S_4_O_6_^2−^) with three possible S–S bond stretching (S1–S2, S2–S3, and S3–S4) at around 230, 270, and 310 cm^−1^; disulfite ions (S_2_O_5_^2−^) with a single S–S stretching signal at 270 cm^−1^ (overlapping with that of S_4_O_6_^2−^); dithionate ions (S_2_O_6_^2−^) with S–S stretching at 288 cm^−1^ and SO_3_ rocking at 320 cm^−1^; dithionite ions (S_2_O_4_^2−^) with S–S stretching at 250 cm^−1^ and SO_2_ twisting at 330 cm^−1^; and thiosulfate ions (S_2_O_3_^2−^) with S–S stretching at 450 cm^−1^. Sulfate ions (SO_4_^2−^) lack S–S signals but they contribute spectral bands in the interval 300–370 cm^−1^ (i.e., related to S–O–S bending). However, such signals strongly overlap with C–C–O bending signals from a variety of other molecules (Wiercigroch et al., 2017), and can hardly serve as markers for sulfate ions. Note that also indoxyl sulfate ions lack strong signals in the low-wavenumber region, but they present a relatively strong ring-breathing signal at around 690 cm^−1^. Some additional contributions of indoxyl sulfate to the recorded spectra are expected at around 635 and 651 cm^−1^ (cf. molecular structure and Raman spectrum of indoxyl sulfate in Supplementary Figure S1) (Elumalai et al., 2014). Indeed, a signal positioned at ~692 cm^−1^ appears as a clear shoulder in all Raman spectra of the *Cnm*^(+)^*Sm* isolates (cf. label in inset to Figure 3a), while it is hardly resolved in those of the *Cnm*^(−)^*Sm* ones (cf. label in inset to Figure 3b) since completely overlapping stronger signals (cf. Figure 3b), whose origin is discussed later in this section.

As anticipated above, ten distinct but partly overlapping signals in the interval 482–613 cm^−1^ represent skeletal vibrational modes from both *GlcN* and *GlcNAc* molecules (cf. Raman spectra from pure compounds in Supplementary Figure S2) (She et al., 1974). These molecules are important constituents of the peptidoglycan structure of *S. mutans* (cf. sub-bands in blue color and labels in inset to Figure 3). Two additional bands located at 210–220 cm^−1^ and ~365 cm^−1^ (cf. arrows in inset to Figure 3a) can be assigned to both mono and polysaccharides (Konorov et al., 2011; Wiercigroch et al., 2017). However, the former signal also include contributions from D–(+)–glucose (Wiercigroch et al., 2017).

**Figure 4:**
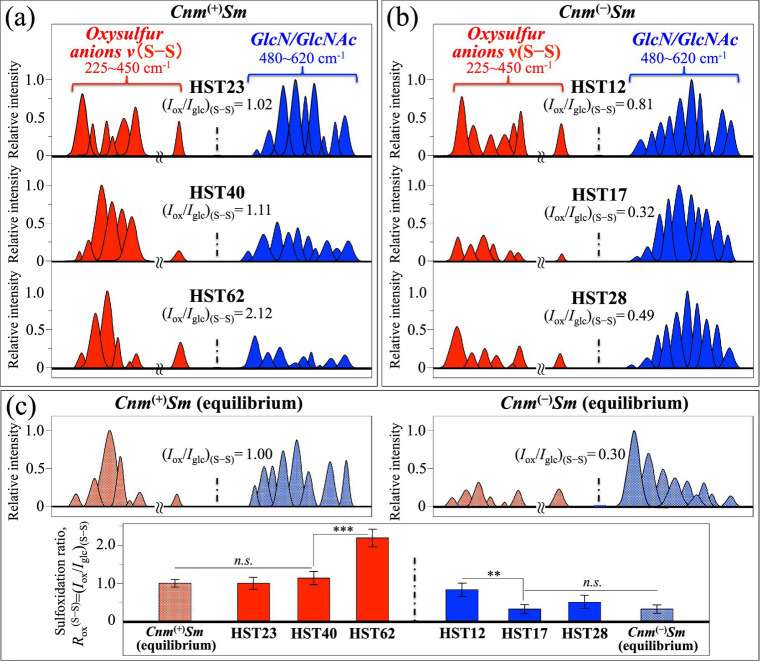
Selected oxysulfur (red) and *GlcN*/*GlcNAc* (blue) signals extracted from the deconvoluted spectra of clinical isolates belonging to **(a)** *Cnm*^(+)^*Sm* and **(b)** *Cnm*^(−)^*Sm* strains (cf. sub-bands from Figures 3a,b, respectively); in inset, computed sulfoxidation ratios, *R*_ox_^**(S–S)**^ = (*I*_ox_/*I*_glc_)_(S–S)_, are shown for each clinical isolate. In **(c)**, two sets of bands with vibrational origins same as above are given with the respective *R*_ox_^(S–S)^ values in inset for both *Cnm*^(+)^*Sm* and *Cnm*^(−)^*Sm* equilibrium strains (as reported in the companion Part I paper) (Pezzotti et al., 2026a) Plots of *R*_ox_^(S–S)^ values and related standard deviations for *Cnm*^(+)^*Sm* and *Cnm*^(−)^*Sm* clinical isolates in comparison with the respective equilibrium strains are also plotted in (c) (*n* = 10; ** = *p* < 10^−2^; *** = *p* < 10^−3^; *n.s.* = non significant).

Now, upon computing the relative intensities of the two sets of Raman signals mainly related to oxysulfur anions (at 229–306 cm^−1^ and 447–450 cm^−1^) and *GlcN*/*GlcNAc* molecules (at 482–613 cm^−1^), a spectroscopic parameter could be conceived, which reflects their respective volumetric impact on the overall bacterial structure. Such spectroscopic parameter was defined as the sulfoxidation ratio, *R*_ox_^(S–S)^ = (*I*_ox_/*I*_glc_)_(S–S)_, in which the cumulative areal intensity covered by signals from oxysulfur anions (red bands) is normalized to the cumulative area of *GlcN*/*GlcNAc* signals (blue bands). Figures 4a,b show the selected oxysulfur (red) and *GlcN*/*GlcNAc* (blue) signals, as extracted from the deconvoluted spectra of clinical isolates belonging to *Cnm*^(+)^*Sm* and *Cnm*^(−)^*Sm* strains, respectively (cf. Figures 3a,b, respectively). In inset, values are shown of the sulfoxidation ratio, *R*_ox_^(S–S)^, as computed for each clinical isolate. The computed *R*_ox_^(S–S)^ values should be compared with those retrieved from spectra of the *Cnm*^(+)^*Sm* and *Cnm*^(−)^*Sm* equilibrium strains, as reported in the companion Part I paper (cf. spectral bands extracted from the original spectra and *R*_ox_^(S–S)^ values in inset to Figure 4c) (Pezzotti et al., 2026a) Figure 4c also compares *R*_ox_^(S–S)^ values and related standard deviations for *Cnm*^(+)^*Sm* and *Cnm*^(−)^*Sm* clinical isolates to the respective equilibrium strains (cf. also labels for data statistical validity shown in inset). The plots in Figure 4c envisage the three following characteristics:

- Equilibrium strains experienced *R*_ox_^(S–S)^ standard ratios quite different to each other, with the ratio of *Cnm*^(+)^*Sm* being about threefold that of the *Cnm*^(−)^*Sm* one (*R*_ox_^(S–S)^ = 1.0 ± 0.1 vs. 0.3 ± 0.1).
- Both *R*_ox_^(S–S)^ equilibrium values represented minima for the respective sample categories, with the *R*_ox_^(S–S)^ values retrieved from clinical isolates being always equal or larger than the equilibrium ones.
- *R*_ox_^(S–S)^ values computed for *Cnm*^(+)^*Sm* clinical isolates were always larger that those retrieved for *Cnm*^(−)^*Sm* ones.

The above three characteristics all hint to structures with a clear preponderance in *GlcN*/*GlcNAc* molecules for *Cnm*^(−)^*Sm* strains in comparison with *Cnm*^(+)^*Sm* ones. The opposite trend was instead recorded for oxysulfur anions. The meaning of these structural trends and the validity of the *R*_ox_^(S–S)^ ratio as a spectroscopic parameter for assessing virulence characteristics will be discussed in detail in the forthcoming Section 4.

**Figure 5:**
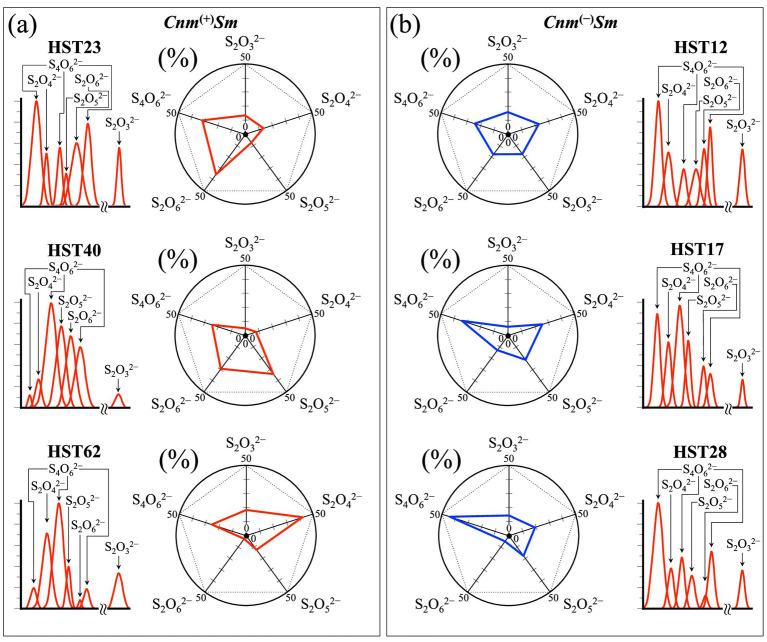
Polar plots of the relative intensities of Raman bands belonging to different oxysulfur compounds for clinical isolates from **(a)** *Cnm*^(+)^*Sm* and **(b)** *Cnm*^(−)^*Sm* strains (cf. labels in inset). Percent values refer to the fractional area of each compound over the total area of the shown sub-bands (extracted from Figure 3).

Figures 5a,b show an attempt to rationalize the results of spectroscopic assessments on different oxysulfur compounds by visualizing the fractional contributions of their respective band intensities in a polar plot for each clinical isolate from *Cnm*^(+)^*Sm* and *Cnm*^(−)^*Sm* sets, respectively (cf. labels in inset). As seen, unlike the *R*_ox_^(S–S)^ ratio, this analysis fails in locating a specific trend that could differentiate *Cnm*^(+)^*Sm* from *Cnm*^(−)^*Sm* isolates. This implies that the distribution of oxysulfur compounds does not obey intrinsic patterns, but it likely depends on the transient metabolic state of each individual strain in its host. This includes the stress state, which could not completely be released upon 24 h culturing in absence of sucrose.

It is known that individual oxysulfur anions play distinct roles in bacterial metabolism (Wu et al., 2021), these anions being involved in various redox processes, particularly in energy generation, sulfur assimilation, and detoxification. Sulfate SO_4_^2−^ ions are the most oxidized form of sulfur and are used by a number of different bacteria in both assimilatory and dissimilatory pathways, the former reducing SO_4_^2−^ to sulfide S^2−^ (to be incorporated in cysteine and methionine) and the latter to be used as terminal electron acceptors in anaerobic respiration upon reduction to hydrogen sulfide H_2_S. Sulfite SO_3_^2−^ ions similarly contribute the dissimilatory pathway being reduced to sulfide, but can also be oxidized back to sulfate to provide an energy source. Thiosulfate S_2_O_3_^2−^ ions serve as both electron donors and electron acceptors in bacterial metabolism. Their oxidation to sulfate or tetrathionate S_4_O_6_^2−^ generates energy, while their reduction to sulfide during dissimilatory sulfur metabolism makes them terminal electron acceptors. The diversity in the use of oxysulfur anions by bacteria highlights their versatility in adapting to different environmental conditions and energy sources. In summary, while the overall amount of oxysulfur anions appears to be an intrinsic characteristic common to all *Cnm*^(+)^*Sm* isolates, the distributions found in the polar plots of different oxysulfur molecules, as shown in Figure 5, might merely represent different physiological stages and balance in metabolic roles of the investigated strains rather than being indicators of their genomic character.

More specifically regarding adhesion, bacteria bind to soft tissues using highly selective lock-and-key strategies complementing receptors on the host surfaces. In streptococci, adhesins are usually composed of proteins in the form of fimbriae (or fibrillae) anchoring lipoteichoic acid and the receptors are the NH_2_ terminals of fibronectin molecules deposited on and bound to epithelial cells (Beachey and Courtney, 1987). Besides the adhesive power of S–S bonds (i.e., among cysteine residues), also S–O interactions are known for their electrostatic aspect, which in turn results in a strong dipolar character with negative charge centered on oxygen. Protonated amino acid terminals could thus directly react with sulfoxide ions or be simply absorbed on them. Indeed, multivalent sulfate ions were already reported to exhibit high binding affinity to collagen molecules by forming bridges between positively charged amino acid residues in regions of high positive charge (Mertz and Leikin, 2004). Although the precise chemical mechanisms exploited by *Cnm*^(+)^*Sm* yet remain to be elucidated, our present findings suggest that oxysulfur molecules are employed by these evolved strains to boost up electrostatic interactions at the initial stage of adhesion to soft tissue. This point will be discussed further in the forthcoming Section 4.2.

Another bold difference spotted in comparing spectra from *Cnm*^(+)^*Sm* and *Cnm*^(−)^*Sm* strains in Figure 3 deals with a doublet located at around 671 and 687 cm^−1^. These bands, which are much more prominent in *Cnm*^(−)^*Sm* strains, could be assigned to symmetric stretching of 6-ring and 5-ring deformation modes, respectively (i.e., in guanine, adenine, and thymine overlapping vibrations), and could thus be traced back to extracellular DNA (eDNA) (Madzharova et al., 2016). As discussed in the companion Part I paper (Pezzotti et al., 2026a), *Cnm*^(−)^*Sm* tends to accumulate eDNA within its built biofilm structure as a result of a multitasking strategy encompassing its origins (Sharma and Rajpurohit, 2024). This includes forming a stable and continuous filamentous network to structurally stabilize the biofilm (Böckelmann et al., 2006; Buzzo et al., 2021), being a source of nutrients (Liao et al., 2014; Serrage et al., 2021), acting as an adhesive factor in both early and consolidating stages of biofilm formation (Herman-Bausier et al., 2017; Secchi et al., 2022), and contributing biofilm resistance against antimicrobial agents (Chiang et al., 2013; Hall and Mah, 2017).

In summary, Raman analyses in Zone I unfolds two so far unreported and, for some aspects, opposite strategies adopted by *Cnm*^(+)^*Sm* and *Cnm*^(−)^*Sm* strains: the former intrinsically alters its structure in order to boost up adhesive properties and to successfully nestle in cardiovascular niches, while the latter mainly aims at defending its own environment and at promoting cell proliferation within stably structured biofilms.

#### 3.4.2 Zone II—intermediate wavenumber region (700–1200 cm^−1^)

**Figure 6:**
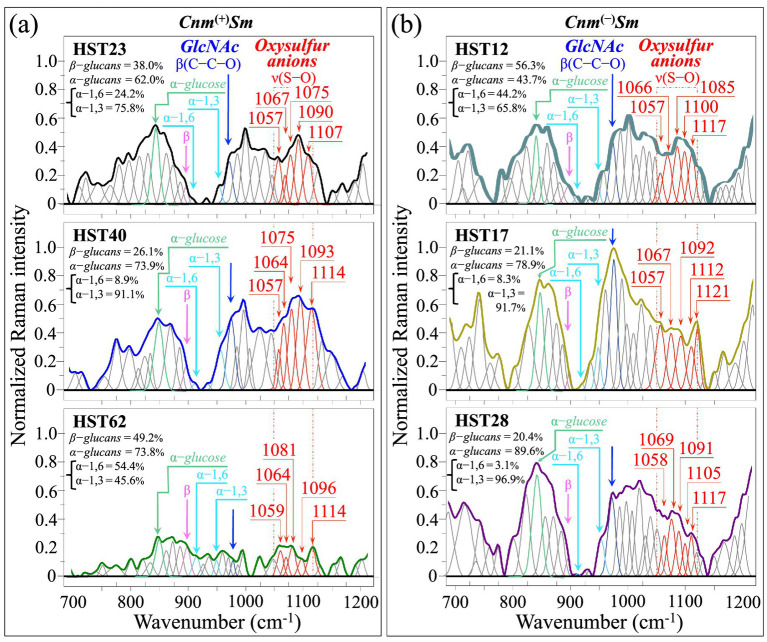
High spectrally resolved (average) Raman spectra in Zone II (700–1200 cm^−1^) as collected on **(a)** *Cnm*^(+)^*Sm* and **(b)** *Cnm*^(−)^*Sm* clinical isolates (cf. labels in inset). Sub-bands emphasized in red represent S–O stretching signals from oxysulfur anions (cf. text). Other signals of interest in this spectral zone are: C–C–O bending in *GlcNAc*, C−O−C bending in *β* − glucans, *α* − 1,6 − and α − 1,3 − linked glucans, and C−C stretching in glucose/sucrose. The computed fractions of different glucans are given in inset to each spectrum. Wavenumbers in correspondence of oxysulfur anion signals are given in cm^−1^.

Figures 6a,b display two series of high spectrally resolved (average) Raman spectra collected on *Cnm*^(+)^*Sm* (HST23, HST40, and HST62; cf. labels in inset) and *Cnm*^(−)^*Sm* (HST12, HST17, and HST28; cf. labels in inset) isolates, respectively. Zone II is important primarily for the presence of stretching signals from S–O bonds, which contributes a series of five distinct, but partly overlapping signals in the wavenumber interval between 1057 and 1121 cm^−1^. The sulfur-containing molecules responsible for the above-mentioned five signals could be identified, as follows (Sato et al., 1985): disulfite ions (S_2_O_5_^2−^) with S–O_2_ or S–O_3_ stretching signals at 1055–1070 cm^−1^; indoxyl sulfate ions with a S–O_2_ stretching signal at ~1055 cm^−1^ (cf. Raman spectrum of indoxyl sulfate in Supplementary Figure S1) (Elumalai et al., 2014); peroxydisulfate ions (S_2_O_8_^2−^) with S–O_3_ symmetric stretching at 1085–1090 cm^−1^; dithionate ions (S_2_O_6_^2−^) with S–O_3_ symmetric stretching at 1090–1095 cm^−1^; disulfate ions (S_2_O_7_^2−^) with S–O_3_ symmetric stretching at ~1065 cm^−1^ (overlapping with S_2_O_5_^2−^); and sulfate ions (SO_4_^2−^) with S–O signals at 1100–1120 cm^−1^. Note that thiosulfate ions (S_2_O_3_^2−^) present their S–O_3_ antisymmetric and symmetric stretching signals at higher wavenumbers (i.e., 1155 and 1130 cm^−1^, respectively) (Sato et al., 1985). Since these signals strongly overlap with C–C skeletal stretching in various molecules and C–N stretching in proteins (Movasaghi et al., 2007), they will be excluded from the following quantitative assessments. Similar considerations hold for the S–O_3_ stretching band of tetrathionate ions (S_4_O_6_^2−^) at ~1050 cm^−1^, which overlaps with signals from both glycogen and proteins (Movasaghi et al., 2007).

**Figure 7:**
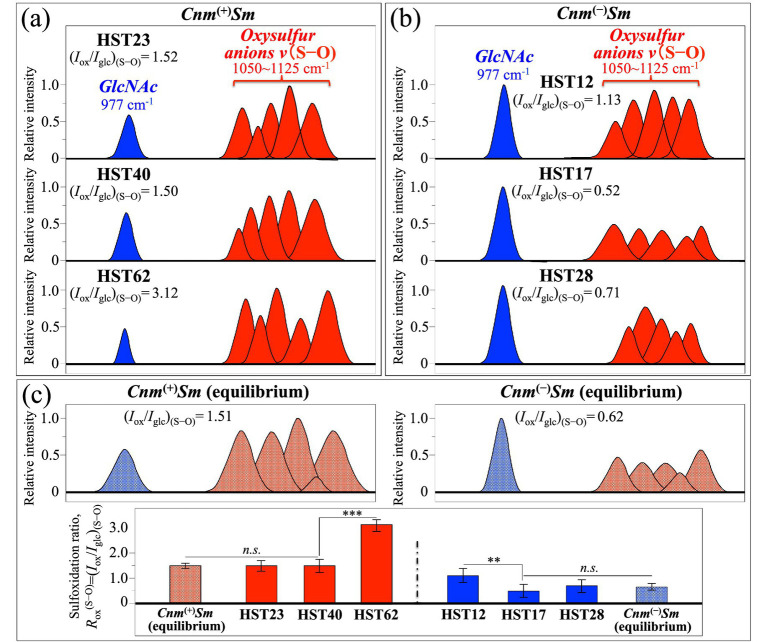
Selected oxysulfur (red) signals and *GlcNAc* (blue) signal extracted from the deconvoluted spectra of clinical isolates belonging to **(a)** *Cnm*^(+)^*Sm* and **(b)** *Cnm*^(−)^*Sm* strains (i.e., as extracted from Figures 6a,b, respectively); values in inset report the computed sulfoxidation ratios, *R*_ox_^**(S–O)**^ = (*I*_ox_/*I*_glc_)_(S–O)_, for each clinical isolate. In **(c)**, two sets of bands with the same vibrational origins as above are given with the respective *R*_ox_^(S–O)^ values (in inset) for both *Cnm*^(+)^*Sm* and *Cnm*^(−)^*Sm* equilibrium strains (as reported in the companion Part I paper) (Pezzotti et al., 2026a) Plots of *R*_ox_^(S–O)^ values and related standard deviations for *Cnm*^(+)^*Sm* and *Cnm*^(−)^*Sm* clinical isolates in comparison with the respective equilibrium strains are also plotted in (c) (*n* = 10; ** = *p* < 10^−2^; *** = *p* < 10^−3^; *n.s.* = non significant).

Similar to the procedure followed above for the S–S bands, the cumulative sum of relative intensities for S–O signals comprised between 1057 and 1121 cm^−1^ (cf. red colored bands in Figure 6) was computed and compared with that arising from *GlcNAc* molecules at ~977 cm^−1^ (C–C–O bending) in the same Zone II (cf. blue colored band in Figure 6). An additional spectroscopic parameter, also referred to as sulfoxidation ratio, *R*_ox_^(S–O)^ = (*I*_ox_/*I*_glc_)_(S–O)_, was defined with the scope of validating the previously defined one, *R*_ox_^(S–S)^ = (*I*_ox_/*I*_glc_)_(S–S)_ (cf. Figure 4c). Figures 7a,b summarizes the outputs of Raman S–O analysis on bacterial isolates by showing oxysulfur bands (red) and *GlcNAc* band (blue) as extracted from the respective spectra in Figure 6, and the computed *R*_ox_^(S–O)^ = (*I*_ox_/*I*_glc_)_(S–O)_ values (in inset). Trends similar to those recorded in analyzing S–S signals were found, with *R*_ox_^(S–O)^ ratios from the *Cnm*^(+)^*Sm* equilibrium strain being ~2.5 higher than that of the *Cnm*^(−)^*Sm* one, and *R*_ox_^(S–O)^ values computed for *Cnm*^(+)^*Sm* clinical isolates always clearly larger that those retrieved for *Cnm*^(−)^*Sm* ones. Interestingly, the values of *R*_ox_^(S–O)^ ratios were systematically higher by a factor ~1.5 than the respective *R*_ox_^(S–S)^ ones, this factor mainly arising from difference in cross section between S–S and S–O Raman signals. In summary, the Raman analysis on oxysulfur molecules performed in Zone II validated that performed for Zone I, thus confirming a preponderance in *GlcN*/*GlcNAc* molecules (over oxysulfur ones) in *Cnm*^(−)^*Sm* strains and the opposite trend for *Cnm*^(+)^*Sm* ones.

In addition to S-related bands, Zone II also included signals from glucans in the region 890–941 cm^−1^ (Wiercigroch et al., 2017). In particular, the bands at ~890, 919, and 941 cm^−1^ represent markers for C−O−C bending in *β* − glucans, *α* − 1,6−, and α − 1,3 − linked glucans, respectively. These bands are emphasized in purple and light blue colors in Figure 6 (cf. labels in inset), while the light-green signal at around 851 cm^−1^, represents C−C stretching (with a minor components of CH_2_ torsion) in both glucose and sucrose rings (Wiercigroch et al., 2017). The fraction of *α* − to β − glucans and, within the α − structure, that of α − 1,6 − and α − 1,3 − linked ones were computed according to algorithms proposed and validated in previous works (Hosoki et al., 2020; Pezzotti et al., 2022a, 2022b, 2022c, 2023b). The above fractions were computed from the Raman spectroscopic ratios *R_α/β_* = (*I*_941_ + *I*_919_)/(*I*_890_ + *I*_944_ + *I*_919_) and *R_α3/α6_* = *I*_944_/*I*_919_. Glucan fractions, which stem from biofilm residues in the original clinical sample, showed widely fluctuating values among different clinical isolates (cf. values in inset to Figures 6a,b). However, a comparison between *Cnm*^(−)^*Sm* and *Cnm*^(+)^*Sm* strains shows that the former ones generally tended to include higher fractions of *α* − over β − glucans and of α − 1,3 − over α − 1,6 − linked glucans. This observation appears to support the trend discussed above suggesting that *Cnm*^(−)^*Sm* strains adopt a more “protective” strategy as compared to *Cnm*^(+)^*Sm* ones, since *α* − 1,3 − linked glucans possess high stiffness and impermeability characteristics among glucans, while also protecting bacterial cells from drugs and degrading enzymes encountered during infection (Fujikawa et al., 2009). Additional consideration will be given in the next Section 3.5, in which glucans signals were collected on biofilms developed by the isolates after culturing them for 2 days with sucrose addition. Nevertheless, the high degree of fluctuation in glucan fractions, as retrieved from their respective Raman markers, did not allow us to reliably adopt glucan signals as markers that differentiate *Cnm*^(−)^*Sm* from *Cnm*^(+)^*Sm* strains.

An additionally important hint for the intrinsically different metabolisms of *Cnm*^(−)^*Sm* and *Cnm*^(+)^*Sm* strains resided in the relative intensity of the 851 cm^−1^ glucose/sucrose band, which was always higher in the former strains. This spectral feature confirms the finding reported in the companion Part I paper, showing that *Cnm*^(−)^*Sm* is capable of storing higher fractions of sugar molecules as a reserve of energy within its highly protective biofilm (Pezzotti et al., 2026a).

#### 3.4.3 Zone III—high wavenumber region (1200–1800 cm^−1^)

**Figure 8:**
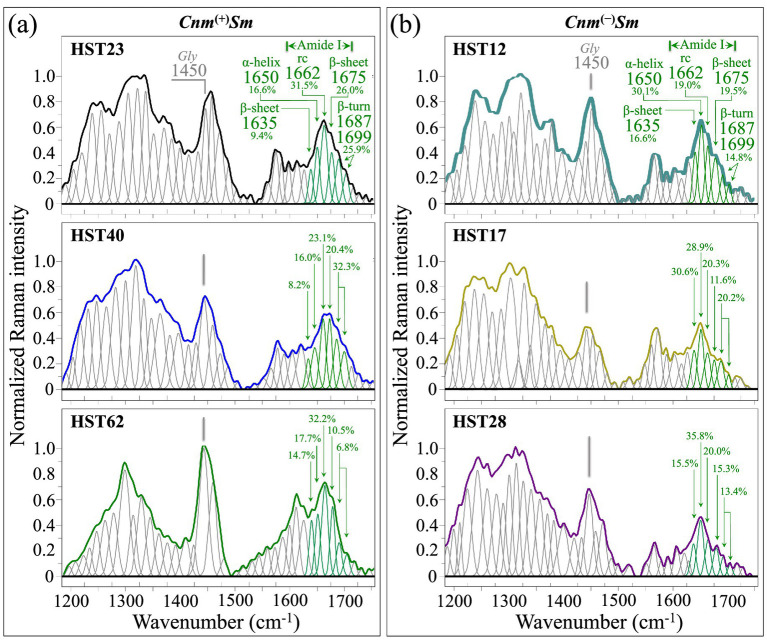
High spectrally resolved (average) Raman spectra in Zone III (1200–1750 cm^−1^) collected on **(a)** *Cnm*^(+)^*Sm* and **(b)** *Cnm*^(−)^*Sm* clinical isolates (cf. labels in inset). Sub-bands emphasized in green represent Amide I signals from secondary structures of proteins (cf. labels in inset). The computed fractions of different secondary structures are given in inset to each spectrum. The band at ~1450 cm^−1^ is mainly contributed by glycine (*Gly*), which is one of the main amino acids composing the *Cnm* protein. Wavenumbers in correspondence of each band are given in cm^−1^.

**Figure 9:**
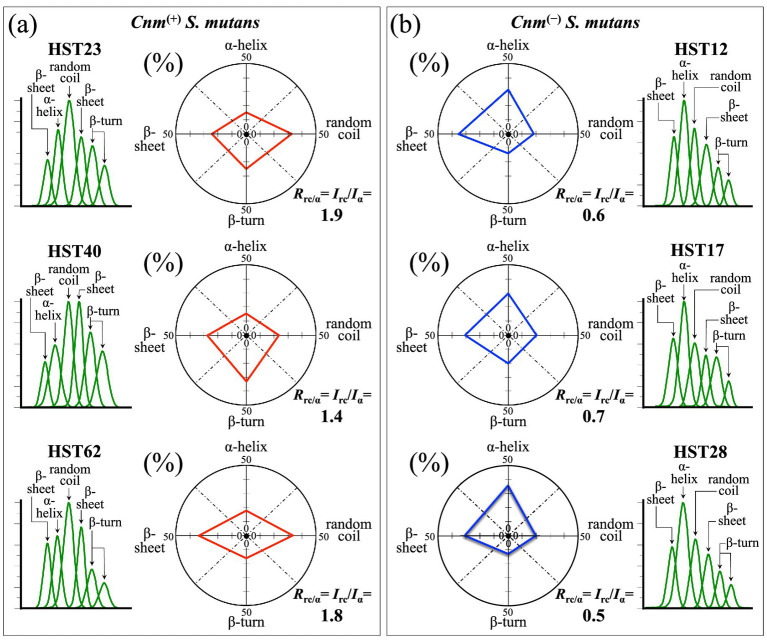
Polar plots of the relative intensities of Raman bands belonging to different secondary structures of proteins belonging to clinical isolates from **(a)** *Cnm*^(+)^*Sm* and **(b)** *Cnm*^(−)^*Sm* strains (cf. labels in inset). Percent values refer to the fractional area of each compound over the total area of all shown sub-bands (extracted from Figure 8). In inset to each plot, the spectroscopic ratios, *R*_rc/α_ = *I*_1660_/*I*_1649_, are given as retrieved from spectral areas subtended under random coil and α-helix signals at ~1660 and ~1649 cm^−1^, respectively. The *R*_rc/α_ ratio could be taken as a measure of the level of disorder in the protein structure of the bacterial isolates, the higher the ratio the higher the disorder.

The most important feature in Zone III (Figure 8) is represented by the Amide I region, related to protein secondary structures and approximately comprised between 1600 and 1700 cm^−1^. In this region, the morphology of Raman spectra from both *Cnm*^(+)^*Sm* and *Cnm*^(−)^*Sm* strains displayed significant differences (cf. Figures 8a,b, respectively). Previous studies have precisely established the spectral locations of bands characteristic of different protein secondary structures: α-helix (at ~1649 cm^−1^), β-sheet (at ~1636 and ~1671 cm^−1^), random coil (at ~1660 cm^−1^), and β-turn (~1684 and ~1695 cm^−1^) (Krimm and Bandekar, 1986; Dong et al., 1990; Sadat and Joye, 2020). Similar to the case of equilibrium strains (Pezzotti et al., 2026a), a quantitative analysis of the area subtended by each Raman band revealed that α-helix was the dominant secondary structure in the protein composition of all *Cnm*^(−)^*Sm* isolates, while the protein structure of all *Cnm*^(+)^*Sm* isolates presented its main structure as random coil (cf. fractional data in inset to Figures 8a,b). In other words, a comparison between *Cnm*^(+)^*Sm* and *Cnm*^(−)^*Sm* isolates pointed to a higher degree of disorder in the former group of samples. As discussed in the companion Part I paper (Pezzotti et al., 2026a), a disordered structure could be considered as a necessary feature in maintaining structural stability in the *Cnm*^(+)^*Sm* bacterial cells, especially in acidic environments (di Cologna et al., 2021). Figures 9a,b summarize in a series of polar plots the protein secondary structures recorded on clinical isolates belonging to *Cnm*^(+)^*Sm* and *Cnm*^(−)^*Sm* categories, respectively. The spectroscopic ratio, *R*_rc/α_ = *I*_1660_/*I*_1649_, retrieved from spectral areas subtended under the random coil and α-helix signals (i.e., at ~1660 and ~1649 cm^−1^, respectively) is given in inset to each plot. The *R*_rc/α_ spectroscopic parameter is a measure of the level of disorder in the protein structure of the bacterial isolate, the higher the ratio the higher the disorder. *R*_rc/α_ ratios computed for *Cnm*^(+)^*Sm* isolates were always two to threefold higher than those recorded on *Cnm*^(−)^*Sm* isolates. This finding quantitatively proves the difference in the degree of protein disorders between the two different sets of samples.

Note that information about protein secondary structure is also encrypted in the Amide III spectral zone between 1220 and 1300 cm^−1^ (cf. also data on equilibrium strains in the companion Part I paper) (Pezzotti et al., 2026a) However, the Amide III zone heavily overlaps with signal contributions from *Cnm* protein residues (e.g., CH_2_ wagging vibrations from glycine backbone and proline side chains, and C–H deformation signals from all proteins). Such overlap, in addition to further overlapping with signals from carbohydrate and glucose structures from biofilm residues, makes it difficult to estimate protein secondary structure in clinical isolates from the relative intensities of Amide III signals. Also related to protein secondary structures is the wavenumber interval 1510–1580 cm^−1^, referred to as the Amide II zone (cf. details in the companion Part I paper) (Pezzotti et al., 2026a) However, similar to the Amide III zone, also the Amide II zone is not suitable for quantitatively assessing protein secondary structures. In particular, the enhancement of two bands at 1565 and 1579 cm^−1^ is contributed by signals of amino acid residues in the *Cnm* motif-anchored protein (cf. Supplementary Information of the companion Part I paper) (Pezzotti et al., 2026a) Moreover, wavenumbers at around 1565 cm^−1^ are also contributed by indoxyl sulfate (cf. Raman spectrum of indoxyl sulfate in Supplementary Figure S1) (Elumalai et al., 2014). One signal that appear to be less affected by overlapping is the band at ~1450 cm^−1^ from glycine (one of the amino acids preponderant in the *Cnm* protein structure) (Pezzotti et al., 2026a) This band, which arises from CH_2_ and N–C–H bending vibrations, indeed appears to be more prominent in the spectra of *Cnm*^(+)^*Sm* isolates (cf. Figures 8a,b).

**Figure 10:**
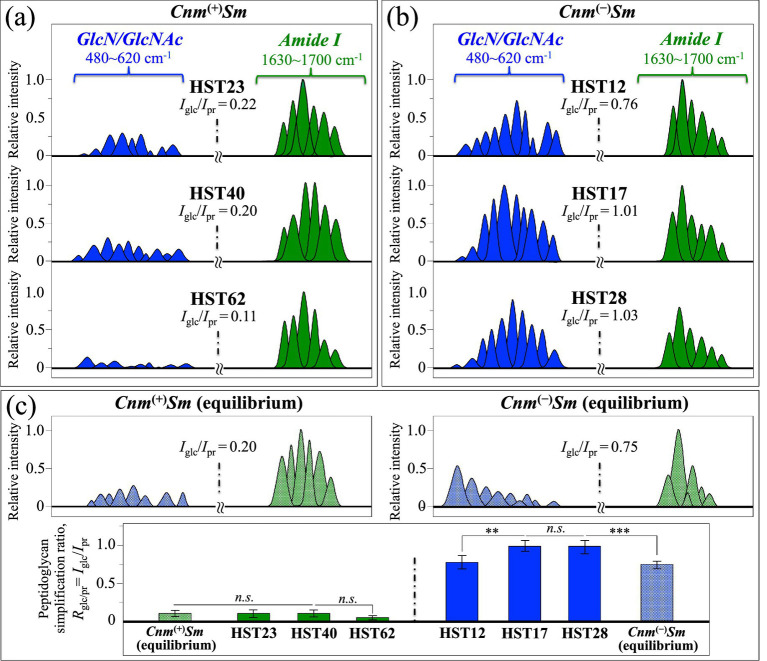
Amide I (from Zone III, Figure 8; green) and *GlcN*/*GlcNAc* (from Zone I, Figure 3; blue) signals, are re-plotted as extracted from deconvoluted high-resolution spectra of clinical isolates belonging to **(a)** *Cnm*^(+)^*Sm* and **(b)** *Cnm*^(−)^*Sm* strains; in **(c)**, results are shown for a similar procedure applied to the same signals retrieved from spectra of the *Cnm*^(+)^*Sm* and *Cnm*^(−)^*Sm* equilibrium strains (cf. companion Part I paper) (Pezzotti et al., 2026a, 2026b). The structural simplification ratio, *R*_glc/pr_ = *I*_glc_/*I*_pr_, is computed and shown in inset for each isolate and equilibrium strain (cf. definition in text). A comparison among *R*_glc/pr_ values is offered in **(c)**, which includes standard deviations (cf. labels in inset for data statistical validity; *n* = 10; ** = *p* < 10^−2^; *** = *p* < 10^−3^; *n.s.* = non significant).

Adopting the Amide I zone as a marker for protein content, we computed from the spectra of each isolate the cumulative (relative) intensities of proteins’ signals, *I*_pr_, and compared them with the respective cumulative intensities, *I*_glc_, of peptidoglycan *GlcN*/*GlcNAc* signals in Zone I (i.e., at 480–620 cm^−1^; the same signals shown in Figure 4). Figures 10a,b show the Amide I (green) and *GlcN*/*GlcNAc* (blue) signals, as extracted from the deconvoluted spectra of clinical isolates belonging to *Cnm*^(+)^*Sm* and *Cnm*^(−)^*Sm* strains, respectively (cf. Figures 3, 8). In section (c) of the same Figure, results are shown for a similar procedure applied to the corresponding signals retrieved from spectra of the *Cnm*^(+)^*Sm* and *Cnm*^(−)^*Sm* equilibrium strains, as reported in the companion Part I paper (Pezzotti et al., 2026a, 2026b) A spectroscopic parameter was then conceived as the peptidoglycan simplification ratio, *R*_glc/pr_ = *I*_glc_/*I*_pr_, namely, the relative reduction in glycan backbone contribution to total cellular protein signal, in which the cumulative areal intensity covered by signals from *GlcN*/*GlcNAc* is normalized to the cumulative area of Amide I signals. This spectroscopic ratio reflects relative glycan-associated Raman signal normalized to total protein and should be interpreted as a compositional indicator rather than a direct structural measurement of peptidoglycan cross-linking or architecture. The *R*_glc/pr_ value thus reflects the relative contribution of glycan backbone-associated Raman signal to total cellular protein signal. While this spectroscopic ratio provides a compositional index of cell wall carbohydrate relative to biomass, it does not directly measure molecular parameters such as cross-linking density or glycan strand length. Therefore, changes in this ratio could also incorporate shifts in relative different glycan contribution in addition to a definitive structural simplification.

The computed values are given in inset to Figures 10a–c. In Figures 10c, a comparison is shown for *R*_glc/pr_ values and related standard deviations as collected on *Cnm*^(+)^*Sm* and *Cnm*^(−)^*Sm* clinical isolates in comparison with the respective equilibrium strains (cf. also labels for data statistical validation in inset). The Raman outputs of protein analyses show that the *R*_glc/pr_ ratio from the *Cnm*^(+)^*Sm* equilibrium strain was almost four times lower than that of the *Cnm*^(−)^*Sm* one. Similarly, the *R*_glc/pr_ ratios retrieved for the *Cnm*^(+)^*Sm* clinical isolates are always 4–5 times lower that those of the *Cnm*^(−)^*Sm* ones.

In summary, Raman analyses in Zone III confirmed that *Cnm*^(+)^*Sm* strains tend to alter (simplify) their peptidoglycan structures while enriching their structure with highly disordered proteins. We suggest that this process is part of an overall strategy aimed at strengthening the bacteria adhesive properties.

### 3.5 Raman spectra of biofilm containing samples

**Figure 11:**
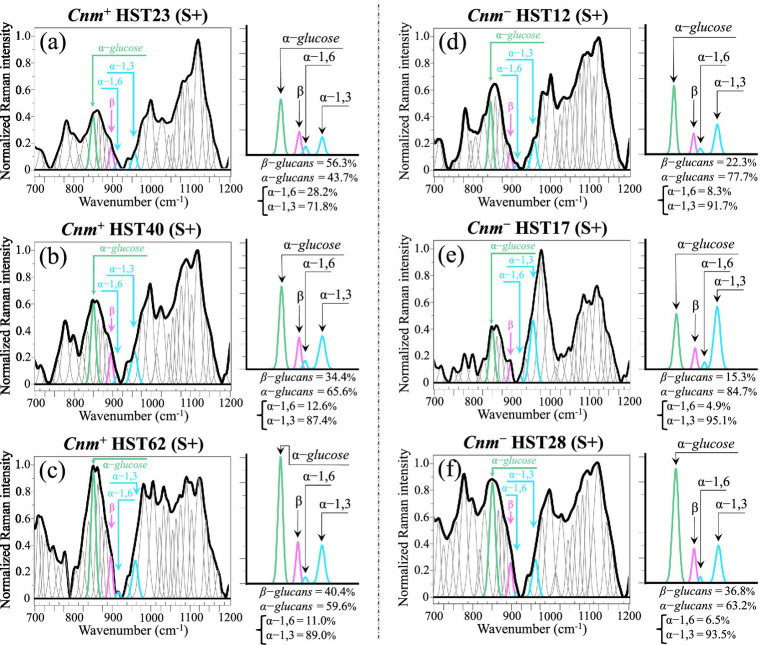
**(a–c)** Average and deconvoluted Raman spectra in Zone II as collected on *Cnm*^(+)^*Sm* isolates, while **(d–f)** give similar spectra for *Cnm*^(−)^*Sm* isolates (cf. labels in inset) cultured for 2 days with addition of sucrose (S+). On the right side of each spectrum, signals at ~890, 919, and 941 cm^−1^ are extracted, which represent markers for β − glucans, α − 1,6 − and α − 1,3 − linked glucans, respectively. The additional signal from α − glucose rings at around 851 cm^−1^ is also shown as a marker for free sugar molecules within the biofilm. Relative fractions for the above molecules, which were computed from the areal fractions subtended by each signal, are explicitly reported in inset.

Figures 11a–c show average and deconvoluted Raman spectra in the region 700–1200 cm^−1^ (Zone II), as collected on samples of *Cnm*^(+)^*Sm* clinical isolates cultured for 2 days with addition of sucrose (cf. labels in inset). Similarly, Figures 11d–f show spectra collected under the same experimental conditions on sample of *Cnm*^(−)^*Sm* clinical isolates (cf. labels in inset). On the right side of each deconvoluted spectrum, deconvoluted signals at ~890, 919, and 941 cm^−1^ were extracted, which represent markers for *β* − glucans, *α* − 1,6 − and α − 1,3 − linked glucans, respectively. The additional signal from α − glucose rings at around 851 cm^−1^ is also reported as a marker for free sugar molecules, which represents the reserve of available energy within the biofilm. The fractions of α − and β − glucans and, within the α − glucan structure, that of α − 1,6 − and α − 1,3 − linked ones were computed according to the same algorithms employed in the previous section for isolates cultured in the absence of sucrose (cf. labels in inset to each section). Data in Figure 11 were collected in an attempt to link spectral signals from glucan fractions that stem from biofilm residues in the original clinical sample to the corresponding signals after bacterial stabilization *in vitro*. The only hint previously obtained from comparing the widely fluctuating fractions of glucans among different clinical isolates cultured without sucrose (cf. Figure 6) was a more marked tendency for the *Cnm*^(−)^*Sm* isolates over the *Cnm*^(+)^*Sm* ones to produce higher fractions of α − over β − glucans and of α − 1,3 − over α − 1,6 − linked glucans. This observation suggested a more “protective” strategy for the *Cnm*^(−)^*Sm* isolates *in vivo* as compared to the *Cnm*^(+)^*Sm* ones, namely, improving both biofilm stiffness and impermeability from drugs and degrading enzymes. Looking now at biofilm data collected after stabilization in sucrose of the bacterial isolates (Figure 11), the high degree of fluctuation in glucan fractions remained, which definitely forestalled the possibility to reliably use glucan signals as markers to differentiate *Cnm*^(−)^*Sm* from *Cnm*^(+)^*Sm* strains. Upon *in vitro* stabilization, both types of isolates appeared to commonly attempt to keep or even increase the fraction of α − 1,3 − above that of α − 1,6 − linked glucans. *Cnm*^(+)^*Sm* isolates approached fractions detected in *Cnm*^(−)^*Sm* samples. Differences in the relative intensity of the 851 cm^−1^ α − glucose rings also seemed to conspicuously disappear. From the data shown in Figure 11, it clearly appears that the biofilm structure of *S. mutans* greatly depends on the pathophysiological state rather than being an intrinsic characteristic of the isolate.

## 4 Discussion

### 4.1 Quantitative spectroscopic parameters for *Cnm*^(+)^*Sm* diagnostics

In a pioneering paper on *S. mutans* adhesion mechanisms, Staat et al. (1980) developed a two-reaction model of adherence in which one initial reaction deals with attachment to the tooth pellicle mediated by cell-surface proteins (rather than glucans or teichoic acids), while a following reaction involves cellular accumulation mediated by sucrose-derived D-glucans and cell surface lectins. This view was in contrast with the traditional view in oral microbiology, according to which *S. mutans* adherence to smooth surfaces is generally considered to depend on the presence of sucrose-derived α − 1,6 − and α − 1,3 − linked water-insoluble D-glucans (i.e., produced by a group of glucosyltransferases). Staat’s research provided for the first time a series of experimental and clinical evidences proving that the cell-surface molecule responsible for sucrose-independent adherence of *S. mutans* is a protein in nature.

About a decade later, Hasty et al. (1992) reported about the expression of multiple adhesins in streptococci (including proteins) and developed a general model to explain how multiple adhesins may function to confer an advantage in the colonization of both hard and soft tissues. Hasty’s study proposed and validated a model of adhesion based on two sequential steps: an initial hydrophobic interaction (mediated by lipoteichoic acid) followed by a successive non-hydrophobic interaction (mediated by specialized proteins). This model was based on the presence of lipoteichoic adhesins capable to interact with hydrophobic probes in *S. mutans*, as first reported by Gibbons and Etherden (1983). In a successive review paper, Takahashi et al. (2013) reported that oral streptococcal adhesins recognize host sialic acid, a circumstance that mediates both bacteria attachment to saliva-coated hydroxyapatite and adhesion to erythrocytes. These adhesins were described as incorporating N-terminal non-repetitive units including both serine-rich regions and a C-terminal cell-wall anchoring domain. A successive important step forward was made by Nomura et al. (2008), who specifically focused on the Cnm protein of the *S. mutans*, sequenced it from 47 different clinical strains, and found that the nucleotide alignment of the collagen-binding domain always remained well conserved. Their findings clearly demonstrated that the presence of the Cnm protein in *S. mutans* increases the risk of dental caries. However, it was also noticed that several (yet a minority of) subjects positive for *Cnm*^(+)^*Sm* recorded low caries scores, which indicated the presence of additional factors in the development of dental caries. Looking at this latter finding in the perspective of the present paper, one could observe that the Nomura’s paper indeed unveiled some limitations for a diagnostic analysis merely based on genomics.

A more recent paper by Esberg et al. (2017) was the first to demonstrate that (protein) adhesin types coincide with distinct biotypes of *S. mutans*. Those researchers classified *S. mutans* sucrose-independent adhesin biotypes to match and predict individual caries development based on both genetic and phenotypic properties. The detection of SpaP B and Cnm (or Cbm) subtypes coincided with caries increment in 5-years-follow up, and their binding to DMBT1 and saliva correlated with individual caries scores. Moreover, bacteria showed a discriminatory ability related to host variables, with increasing genes encoding of bacterial proteins as a consequence of host adaptation. The SpaP B and Cnm/Cbm biotypes were reported to directly bind to DMBT1 via non-leucine-rich repeats (Loimaranta et al., 2009). Such unequivocal links between adhesin subtypes of *S. mutans* and levels of caries development, as well as the observed genetic effects of host adaptation, could partly explain the out-of-trend cases recorded by Nomura et al. (2008) and represent a first step in developing individualized oral care.

**Figure 12:**
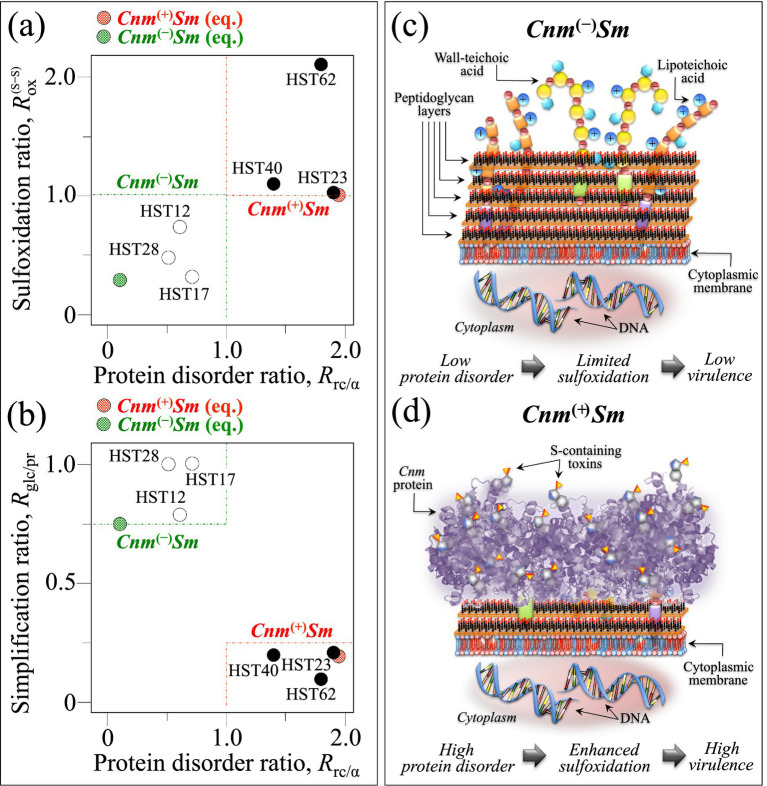
The spectroscopic parameters sulfoxidation ratio, *R*_ox_^(S–S)^, and structural simplification ratio, *R*_glc/pr_, are plotted as a function of the protein disorder ratio, *R*_rc/α_, in **(a,b),** respectively (cf. also data for equilibrium strains are shown for comparison) (Pezzotti et al., 2026a, 2026b) Values related to *Cnm*^(+)^*Sm* and *Cnm*^(−)^*Sm* lie in different quadrants for both plots. They show that the higher the protein disorder the higher the degree of sulfoxidation and the higher the level of peptidoglycan structural simplification. The plot in **(b)** suggests a path to quantify bacterial virulence, the lower *R*_glc/pr_ ratio the higher the attitude of the strain to adhere soft tissue and thus its virulence. The process of peptidoglycan “simplification” of the *Cnm*^(+)^*Sm* structure with respect to the *Cnm*^(−)^*Sm* one is schematically visualized in **(c,d)** (cf. labels in inset).

In the companion Part I paper (Pezzotti et al., 2026a, 2026b), we followed a molecular-medicine approach to match genomics in assessing *S. mutans* virulence. We showed Raman data that unequivocally located molecular structures richer in disordered proteins and oxysulfur compounds, and poorer in peptidoglycan molecules in *Cnm*^(+)^*Sm* as compared to *Cnm*^(−)^*Sm*. Data on clinical isolates, as presented in the present paper, suggest that, although this trend shows some degree of fluctuation, it is basically unaffected by other genetic characteristics. Moreover, the present data seem to confirm the hypothesis of an additional (and yet unreported) adhesion mechanism in *Cnm*^(+)^*Sm* based on sulfur chemistry. Such bold differences in molecular structure give us a chance to propose a Raman spectroscopic path in fast screening between *Cnm*^(+)^*Sm* and *Cnm*^(−)^*Sm* clinical isolates, provided that suitable parameters could be retrieved from statistically significant average Raman spectra. In such an attempt, Figure 12 aims at rationalizing the Raman data presented in Section 3. In sections (a) and (b) of this figure, two spectroscopic parameters, namely, the sulfoxidation ratio, *R*_ox_^(S–S)^, and the structural simplification ratio, *R*_glc/pr_, are plotted as a function of the protein disorder ratio, *R*_rc/*α*_, respectively. Data retrieved from equilibrium strains, as computed from the respective spectra in Part I, are also shown for comparison. As seen, in both plots, values related to *Cnm*^(+)^*Sm* and *Cnm*^(−)^*Sm* lie in different quadrants, the higher the protein disorder (higher *R*_rc/α_ values) the higher the degree of sulfoxidation (higher *R*_ox_^(S–S)^ or *R*_ox_^(S–O)^ values) and the higher the level of peptidoglycan structural simplification (lower *R*_glc/pr_ values). The *R*_glc/pr_ vs. *R*_rc/α_ relationship is particularly striking, categorizes a balance between peptidoglycans and surface Cnm proteins (cf. schematic drafts in Figures 12c,d), and suggests a path to quantify bacterial virulence, the lower the *R*_glc/pr_ ratio the higher bacterial virulence. The trend in (a) additionally suggests that intrinsically disordered proteins are the enclave hosting sulfoxide molecules, thus possibly linking the *R*_ox_^(S–S)^ (and *R*_ox_^(S–O)^) ratio to the adhesion capability of the *S. mutans* strains. Note that the *Cnm*^(+)^*Sm* and *Cnm*^(−)^*Sm* equilibrium strains, while showing the highest and the lowest *R*_rc/α_ values, respectively, only represent minima in both degrees of sulfoxidation and structural simplification as compared to bacterial isolates. This suggests that an increasing state of stress for bacteria in clinical isolates (e.g., due to their prolonged exposure to reactive oxygen species (ROS)) actually exacerbates their structural characteristics towards increased levels of virulence. Since sulfoxides occur as products of the S-oxygenation in proteins and their formation is often associated with oxidative stress, higher *R*_ox_^(S–S)^ and *R*_ox_^(S–O)^ ratios should reasonably correspond to higher stress levels in the host (Kappler et al., 2019).

In summary, provided that the above considerations could be confirmed on a statistically significant number of clinical isolates, the three spectroscopic parameters *R*_ox_^(S–S)^ (or *R*_ox_^(S–O)^), *R*_glc/pr_, and *R*_rc/α_ could become as many virulence indicators. An attempt to clinically validate the Raman approach to a quantitative assessment of *S. mutans* virulence will be presented in the companion Part III paper (Pezzotti et al., 2026b).

### 4.2 Interpreting the sulfoxidation strategy of *Cnm*^(+)^*Sm* strains

A number of bacterial pathogens utilize sulfur compounds, either as part of their metabolic processes or as components that help in pathogenesis (Koch and Dahl, 2018; Kies and Hammer, 2022). While oxysulfur compounds themselves are not generally reported as primary virulence factors in most bacteria, they can play significant intermediate roles in several contexts, such as sulfur metabolism (in pathogenesis) and bacterial adhesion (Wang et al., 2023, 2024). Regarding the former context, certain bacterial pathogens can metabolize sulfur-containing compounds, including oxysulfur compounds like sulfate (SO_4_^2−^), sulfite (SO_3_^2−^), or thiosulfate (S_2_O_3_^2−^), as part of their energy acquisition. For example, pathogens such as *Helicobacter pylori*, which infect the stomach, produce hydrogen sulfite (H_2_S) via cysteine and methionine desulfhydration and use it to generate energy; this process allows bacteria to survive in challenging environments like the acidic gastric mucosa (Lee et al., 2006). Some other bacteria, like *Salmonella enterica*, can use sulfur oxidation pathways that involve thiosulfate as part of their competitive strategy to outcompete gut microbiota in inflamed tissues, thereby aiding colonization during infection (Hinsley and Berks, 2002; Stoffels et al., 2012). *Escherichia coli* uses dimethyl sulfoxide (DMSO) as an electron acceptor in anaerobic respiration (Sambasivarao and Weiner, 1991). The reduction of DMSO produces dimethyl sulfide (DMS) and, during this process, sulfoxide intermediates and potentially sulfoxide anions can form. This process is facilitated by enzymes such as DMSO reductase, which catalyzes the reduction of DMSO to DMS. In the reverse oxidation process, sulfoxide anions might briefly appear. *Porphyromonas gingivalis* exploits sulfur chemistry by filling its nanostructured ceramide exosomes with oxysulfur ions in pursuing a self-defense strategy. Such poisoning exosome structures cause the abrupt and extensive formation of *β*-amyloid fibrils in neuroblastoma cell cultures and might contribute to the development of Alzheimer disease (Pezzotti et al., 2023a). Some other bacteria, particularly those involved in sulfur metabolism (e.g., *Thiobacillus* and *Beggiatoa*), can extensively oxidize sulfur compounds as part of their energy production processes, this process resulting in pH values as low as 1.0 upon their growth (Jones and Santini, 2023). In these latter bacteria, sulfur compounds such as H_2_S can be sequentially oxidized to form intermediates, including sulfoxide compounds and sulfoxide anions.

In the more specific context of adhesion properties, which are in focus here, this study suggests that pathogenic bacteria might modify their surface molecules with sulfur-containing groups, including sulfonate or sulfate groups in order to enhance adhesion to host tissues. This might be especially relevant in pathogenic bacteria that interact with mucosal surfaces or extracellular matrices, which have glycoproteins likely forming interactions with sulfonated bacterial surface molecules (Beachey et al., 1988). Sulfoxide anions can form in bacteria primarily through oxidation reactions involving sulfur-containing amino acid residues, in particular, methionine, whose thioether group (R–S–CH_3_), is promptly oxidized to methionine sulfoxide (R–SO^–^CH_3_) in the presence of ROS and thus acts as a “repair system” in adverse environmental circumstances. These reactions typically occur as part of metabolic processes or in response to environmental oxidative stress. In some cases, under highly oxidative conditions or with the involvement of specific enzymes, oxidation could proceed to a more reactive sulfoxide anion state (R–SO^−^). However, in the majority of biological systems, sulfoxide anions are usually transient and quickly processed further or reduced back to methionine through methionine sulfoxide reductases to protect cells from oxidative damage. A review of the published literature suggests that in many pathogens the presence of oxysulfur compounds may not be a central virulence factor, but their transient presence can participate in bacterial energy metabolism, adhesion, and survival strategies, thus contributing pathogenicity in different environments.

As discussed in Section 4.1, *S. mutans* is generally reported to primarily exploit carbohydrate metabolism and acid production for its pathogenicity, rather than oxysulfur chemistry (Krzyściak et al., 2014). However, it is already known that sulfur compounds play indirect roles in its biology. Like most bacteria, *S. mutans* requires sulfur-containing amino acids (cysteine and methionine) for protein synthesis and growth. These amino acids are involved in cellular processes such as redox balance (through glutathione) and enzyme function (Van Loi et al., 2015). Although these processes do not directly relate to oxysulfur chemistry, sulfur is essential to *S. mutans* for its basic metabolism. Sulfur compounds, particularly in the form of thiol-containing molecules (i.e., cysteine or glutathione), are involved in *S. mutans* ability to resist oxidative stress. The production of ROS occurs as part of immune system defense and induces stress on the bacteria. Environmental stress can be counteracted by sulfur chemistry, which is thus critical for survival in the oral cavity where strong oxidative conditions arise. While oxysulfur molecules like sulfite and sulfate are known agents in detoxification pathways of bacteria, there has so far been limited evidence of *S. mutans* using such pathways directly for specific purposes. The majority of literature on both *Cnm*^(+)^*Sm* and *Cnm*^(−)^*Sm* strains refer to the production of extracellular polysaccharides (such as glucans) and lactic acid from sugar metabolism. The companion Part I paper confirmed these notions and also suggested an important difference between Cnm-positive and Cnm-negative strains (Pezzotti et al., 2026a, 2026b): the former employed all its energy and molecular resources in promptly building a stiff and impermeable biofilm structure, while the latter opted to accumulate part of its molecular resources to store glycogen as an energy source to boost bacterial proliferation.

While biofilm formation and biofilm-based adhesion have so far been considered as the primary mechanisms for virulence (Lemos et al., 2019), no direct evidence was yet reported that *S. mutans* exploits oxysulfur compounds to enhance adhesion. Virulence factors have been primarily described in terms of carbohydrate processing, acid tolerance, and biofilm development. The present work suggests for the first time that *Cnm*^(+)^*Sm* strains might exploit oxysulfur chemistry as a central part of their pathogenic strategy. We speculate here that they keep and recycle oxysulfur molecules for adhesion purposes. In other words, besides sulfur-containing molecules being essential for general metabolism and oxidative stress defense, we speculate that oxysulfur compounds might also play an additional significant role in adhesion and virulence of *Cnm*^(+)^*Sm* strains.

### 4.3 Raman spectroscopy for chair-side/real-time analyses of oral flora

From a general perspective, the present study provides new evidence supporting the potential use of Raman spectroscopy in chair-side applications for real-time analyses of oral flora. Raman spectroscopy has already been reported as a unique tool to identify different bacterial species based on their molecular fingerprints (Lin et al., 2019; Chisanga et al., 2020; Wang K. et al., 2020; Zhang et al., 2022). We took here a further step forward in the direction of Raman oral diagnostics and demonstrated that its use could be extended to analyze the virulence of different strains of the same bacterium, *S. mutans*, and to gain straightforward information about its potential virulence beyond oral caries.

The non-destructive nature of Raman spectroscopy assessments together with the unnecessariness of labels or dyes to detect microorganisms make Raman measurements compatible with life and gives the possibility to follow up metabolic changes and to screen structural variations of living bacteria and other microorganisms in response to drugs, presence of competing strains, or other environmental factors (Kloss et al., 2013; Pezzotti, 2021; Pezzotti et al., 2021a, 2022a, 2023b; Rodriguez et al., 2023). However, while the Raman measurement itself might only take few minutes to be performed and requires minimum sample preparation, bacterial isolation from swabs or biofilm samples might be more laborious and time consuming. The oral cavity is indeed a complex environment including a variety of microorganisms, saliva, and tissues, all of which can contribute to the overall Raman signal detected, making it quite complex the procedure of distinguishing among overlapping signals from a variety of different molecules. In this perspective, two different approaches can be followed. From the one side, specific bacteria could be separated from the swab or biofilm samples retrieved from the patient according to specific procedures. For example, the present study has exploited a standard biofilm separation procedures and applied a short-term sucrose-free culture to *S. mutans* isolates with an overall sample preparation time of about 24 h. Research is presently ongoing to develop faster sample preparation procedures. An alternative approach resides in developing advanced deep-learning algorithms that could be trained to identify and extract key features from Raman spectra that are indicative of specific molecules or microorganisms (Ho et al., 2019). Multivariate and neural network analyses could also be applied to correlate multiple spectral features with chemical concentrations or biological conditions, improving the robustness and reliability of the Raman analyses (Lopes Guimaraes et al., 2018; Wang K. et al., 2020; Sui et al., 2022; Zhang et al., 2022; Zhou et al., 2022). Such multifunctional approaches could help to create a comprehensive database of Raman spectral fingerprints for specific oral bacteria, which could then be used for rapid identification and classification in oral clinical diagnostics.

In summary, the present Raman study opens a new path to real-time analyses of *S. mutans* virulence in a chair-side setting by proving for the first time that *Cnm*^(+)^*Sm* strains could unequivocally be distinguished from *Cnm*^(−)^*Sm* ones according to bold differences in molecular structure. Once validated through a statistically significant series of clinical data, this finding will allow for an immediate interpretation, decision-making, and user-friendly Raman approach to diagnosing the risks of caries, endocarditis, and hemorrhagic strokes. A first step towards such statistical validation will be described in the companion Part III paper.

## 5 Conclusion

A series of six *S. mutans* clinical isolates was analyzed by Raman spectroscopy after biofilm purification and 24 h culture in absence of sucrose. Preliminary genomic analyses clarified that three isolates were Cnm-positive while the remaining three were Cnm-negative. Data were compared with genomically controlled Cnm-positive and Cnm-negative *S. mutans* reference strains obtained from long-term cultures in absence of sucrose and referred to as “equilibrium” strains. Statistically representative Raman spectra were obtained upon collecting and averaging ten spectra at different locations over a total area of ~2 mm^2^ for each bacterial strain. Despite the complexity of the spectra collected, it was possible to locate specific characteristics, which differentiated Cnm-positive from Cnm-negative bacteria, independent of biotypes. The main characteristics could be summarized, as follows:

1. Cnm-positive strains were significantly richer in oxysulfur anions, including disulfite, dithionite, thiosulfate, dithionate, tetrathionate, and indoxyl sulfate. This trend was revealed by the higher relative intensities of the characteristic S–S and S–O stretching bands, detected in the wavenumber intervals 229–306/447–450 cm^−1^ and 1057–1121 cm^−1^, respectively.
2. A comparison between the relative intensities of Amide I signals from proteins (1630–1700 cm^−1^) and skeletal mode signals from peptidoglycan structures (482–613 cm^−1^) revealed a skewed balance towards protein-rich spectra in Cnm-positive isolates.
3. The protein secondary structure of Cnm-positive isolates was always much more disordered than that of Cnm-negative ones, with a preponderance of random coil over *α*-helix structures.

All three above characteristics were in agreement with the respective trends recorded on equilibrium strains, as described in the companion Part I paper.

On the other hand, high diversity and variability were found in the glucan structures of biofilm residues, as a consequence of different biotypes and a variety of host-specific factors including physiological stress, presence of competing bacteria, and possible oral care procedures. This latter circumstance hampers any Raman attempt to distinguish between Cnm-positive and Cnm-negative strains in clinical isolates upon simply screening their biofilm structures. However, three spectroscopic parameters could be located, namely, the sulfoxidation ratio, *R*_ox_^(S–S)^ (or *R*_ox_^(S–O)^), the peptidoglycan simplification ratio, *R*_glc/pr_, and the protein disorder ratio, *R*_rc/α_, which well performed as powerful indicators of the structural differences between Cnm-positive and Cnm-negative strains. Linking these three parameters to each other revealed that a higher amount of protein in the bacterial structure always matched a lower amount of peptidoglycans, and higher disorder in protein secondary structure was always accompanied by a more massive presence of oxysulfur ions. The overall Raman view suggests that Cnm-positive bacteria tend to modify their peptidoglycan structure in favor of an external layer of highly disordered and sulfoxide-ion-rich proteins, in order to exploit sulfur chemistry to maximize their adhesive capacity. At this stage, the link between the peculiar sulfur-related Raman signals in the spectrum of Cnm-positive *S. mutans* and an enhanced adhesion capacity is just an hypothesis, which needs to be experimentally tested in forthcoming studies. Additional analyses on a large number of isolates from human patients will be presented in the companion Part III paper (Pezzotti et al., 2026b) in order to substantiate the statistical relevance of the Raman findings described in this study.

## Data Availability

The original contributions presented in the study are included in the article/Supplementary material, further inquiries can be directed to the corresponding author/s.

## Footnotes

1 Available online at: https://github.com/tseemann/shovill.
