# Supplementary material for: Raman signatures of Cnm-positive Streptococcus mutans: II, screening the virulence of clinical isolates
Source: Front Microbiol. 2026 Apr 22;17:1784126. doi: 10.3389/fmicb.2026.1784126 (PMC13148223; doi:10.3389/fmicb.2026.1784126)
Supplement: Supplementary file 1 [file Image_1.pdf]

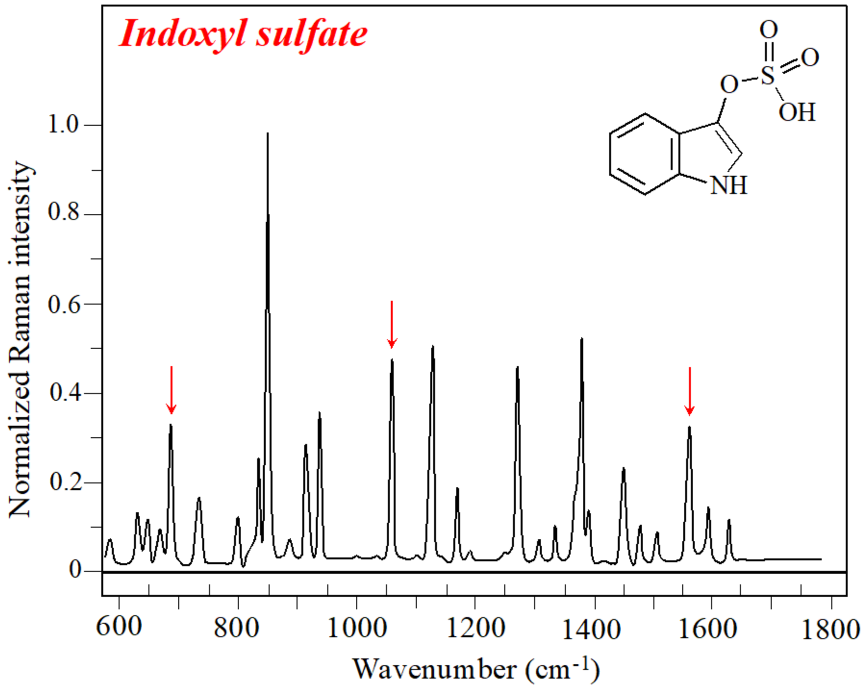

Figure S1: Reference Raman spectrum of indoxyl sulfate (replotted from Ref. 44 as indicated the main text). Arrows indicate bands that could be identified as markers for the indoxyl molecules in the Raman spectra of *S. mutans* clinical isolates.
