# Supplementary material for: Raman signatures of Cnm-positive Streptococcus mutans: II, screening the virulence of clinical isolates
Source: Front Microbiol. 2026 Apr 22;17:1784126. doi: 10.3389/fmicb.2026.1784126 (PMC13148223; doi:10.3389/fmicb.2026.1784126)
Supplement: Supplementary file 2 [file Image_2.pdf]

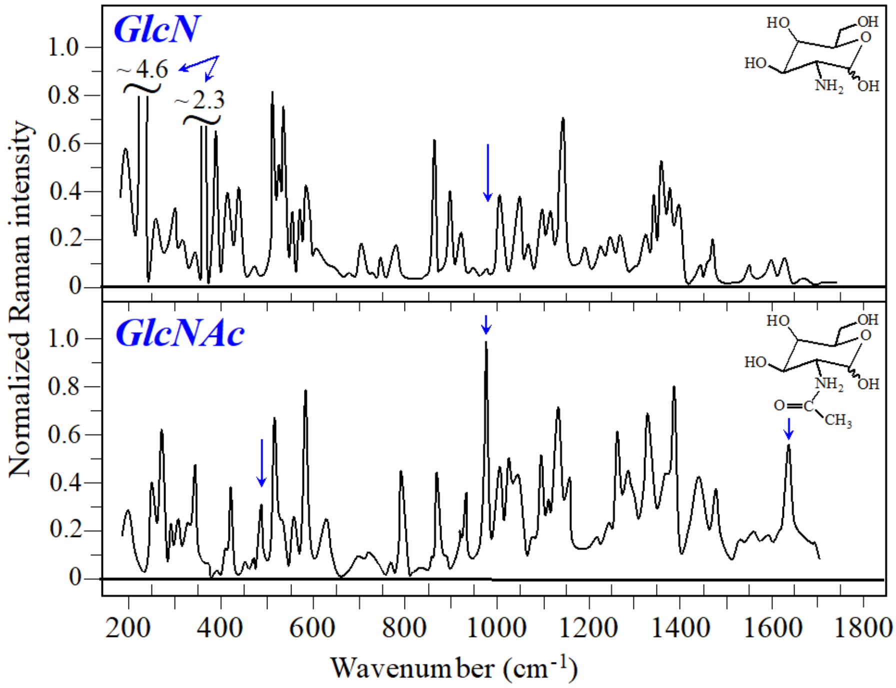

Fig. S2: Reference Raman spectra of glucosamine (*GlcN*) and *N*-acetylglucosamine (*GlcNAc*) molecules (replotted from Ref. 40 as indicated in the main text). Arrows indicate signals that could be used as markers for *GlcNAc* since conspicuously absent in the spectrum of *GlcN*.
