# Supplementary material for: Raman signatures of Cnm-positive Streptococcus mutans: II, screening the virulence of clinical isolates
Source: Front Microbiol. 2026 Apr 22;17:1784126. doi: 10.3389/fmicb.2026.1784126 (PMC13148223; doi:10.3389/fmicb.2026.1784126)
Supplement: Supplementary file 3 [file Table_1.docx]

| **Bacterial species** | **Characteristics** | **Serotype** | ***Cnm* gene** | ***gtf* gene** | | | **Virulence factor** | **Origin** |
| --- | --- | --- | --- | --- | --- | --- | --- | --- |
| ***Streptococcus mutans*** |  |  |  | ***gtfB*** | ***gtfC*** | ***gtfD*** | ***PAc*** |  |
| **HST23** | **clinical isolate** | **c** | **positive** | **positive** | **positive** | **positive** | **positive** | **This study** |
| **HST40** | **clinical isolate** | **c** | **positive** | **positive** | **positive** | **positive** | **positive** | **This study** |
| **HST62** | **clinical isolate** | **c, k** | **positive** | **positive** | **positive** | **positive** | **positive** | **This study** |
| **HST12** | **clinical isolate** | **c** | **none** | **positive** | **positive** | **positive** | **positive** | **This study** |
| **HST17** | **clinical isolate** | **c** | **none** | **positive** | **positive** | **positive** | **positive** | **This study** |
| **HST28** | **clinical isolate** | **c** | **none** | **positive** | **positive** | **positive** | **positive** | **This study** |

**Table I:** Results of whole genome sequence analysis on the investigated six clinical isolates.
